# Ginsenosides, potential TMPRSS2 inhibitors, a trade-off between the therapeutic combination for anti-PD-1 immunotherapy and the treatment of COVID-19 infection of LUAD patients

**DOI:** 10.3389/fphar.2023.1085509

**Published:** 2023-03-13

**Authors:** Mei Meng, Rui Gao, Zixue Liu, Fengxiang Liu, Shiyu Du, Yizhi Song, Jian He

**Affiliations:** ^1^ State Key Laboratory of Oncogenes and Related Genes, Center for Single-Cell Omics, School of Public Health, Shanghai Jiao Tong University School of Medicine, Shanghai, China; ^2^ CAS Key Laboratory of Bio-Medical Diagnostics, Suzhou Institute of Biomedical Engineering and Technology, Chinese Academy of Sciences, Suzhou, China; ^3^ Engineering Laboratory of Nuclear Energy Materials, Ningbo Institute of Materials Technology and Engineering, Chinese Academy of Sciences, Ningbo, China; ^4^ School of Materials Science and Engineering, China University of Petroleum (East China), Qingdao, China; ^5^ School of Computer Science, China University of Petroleum (East China), Qingdao, China

**Keywords:** TMPRSS2, LUAD, biomarker, tumor-infiltrating lymphocytes, prognosis, ginsenosides

## Abstract

**Background:** Acting as a viral entry for coronavirus to invade human cells, TMPRSS2 has become a target for the prevention and treatment of COVID-19 infection. Before this, TMPRSS2 has presented biological functions in cancer, but the roles remain controversial and the mechanism remains unelucidated. Some chemicals have been reported to be inhibitors of TMPRSS2 and also demonstrated other pharmacological properties. At this stage, it is important to discover more new compounds targeting TMPRSS2, especially from natural products, for the prevention and treatment of COVID-19 infection.

**Methods:** We analyzed the correlation between TMPRSS2 expression, methylation level, overall survival rate, clinical parameters, biological process, and determined the correlation between TMPRSS2 and tumor-infiltrating lymphocytes in the tumor and adjacent normal tissue of adenocarcinoma (LUAD) and squamous cell carcinoma (LUSC) respectively by using various types of bioinformatics approaches. Moreover, we determined the correlation between TMPRSS2 protein level and the prognosis of LUAD and LUSC cohorts by immunohistochemistry assay. Furthermore, the cancer immunome atlas (TCIA) database was used to predict the relationship between the expression of TMPRSS2 and response to programmed cell death protein 1 (PD-1) blocker immunotherapy in lung cancer patients. Finally, the putative binding site of ginsenosides bound to TMPRSS2 protein was built from homology modeling to screen high-potency TMPRSS2 inhibitors.

**Results:** We found that TMPRSS2 recruits various types of immunocytes, including CD8^+^, CD4^+^ T cells, B cells and DCs both in LUAD and LUSC patients, and the correlation between TMPRSS2 expression and CD8^+^ and CD4^+^ T cells are stronger in LUAD rather than in LUSC, but excludes macrophages and neutrophils in LUAD patient cohorts. These might be the reason that higher mRNA and protein levels of TMPRSS2 are associated with better prognosis in LUAD cohorts rather than in LUSC cohorts. Furthermore, we found that TMPRSS2 was positively correlated with the prognosis in patient nonresponse to anti-PD-1 therapy. Therefore, we made an inference that increasing the expression level of TMPRSS2 may improve the anti-PD-1 immunotherapy efficacy. Finally, five ginsenosides candidates with high inhibition potency were screened from the natural chemical library to be used as TMPRSS2 inhibitors.

**Conclusion:** All these may imply that TMPRSS2 might be a novel prognostic biomarker and serve as a potential immunomodulator target of immunotherapy combination therapies in LUAD patients nonresponse to anti-PD-1 therapy. Also, these findings may suggest we should pay more attention to LUAD patients, especially those infected with COVID-19, who should avoid medicating TMPRSS2 inhibitors, such as ginsenosides to gain prophylactic and therapeutic benefits against COVID-19.

## 1 Introduction

As a major cause of death from tumor all over the world, lung cancer is mainly induced by genetics, environmental factors, age, gender, unreasonable diet, smoking, and other factors (Gao et al., 2022; He et al., 2023). The primary categories of lung malignant tumor are small cell lung cancer (SCLC) and non-small cell lung cancer (NSCLC). Among them, NSCLC is mainly subdivided into adenocarcinoma (LUAD) and squamous cell carcinoma (LUSC) (He et al., 2021a; Li et al., 2021). At present, compared with previous chemotherapy and surgical treatment, more and more targeted therapy and immune checkpoint inhibitor therapy are applied to the treatment of cancer and have become essential for the treatment of lung cancer treatment (Tan and Tan, 2022). Among them, inhibition of the PD-1 axis, including antibody-mediated programmed cell death protein 1 (PD-1) and programmed cell death ligand 1 (PD-L1) blockade, increased the survival rates of many NSCLC patients (Larsen et al., 2019; He et al., 2022; Tan and Tan, 2022). However, immune drugs benefit only a small percentage of patients and poor prognosis is still the leading cause of a high mortality. Therefore, it is indeed necessary to find a factor or modulator that affects the prognosis of lung cancer to assist the existing treatment methods to improve patients’ prognosis. Additionally, emerging evidence shows that tumor-infiltrating lymphocytes (TIL), such as tumor-infiltrating neutrophils (TIN) and tumor-associated macrophages (TAM), play a critical role in mediating the response to chemotherapy and achieving better clinical outcomes of different cancers (Stanton and Disis, 2016; Su et al., 2021), and have a great effect on the prognosis of cancer (Benevides et al., 2015; Tariq et al., 2017). So, it is also urgently required to elucidate tumor-immune interactions immunophenotypes, as well as identify novel immunological targets for cancer therapies.

TMPRSS2 encodes a serine protease that contains a receptor type A domain, a type II transmembrane domain, a cysteine-rich scavenger receptor domain, and a protease domain. Serine proteases are known to be involved in a wide range of biological processes (Glowacka et al., 2011). It has been found that TMPRSS2 plays a prominent role in COVID-19 infection. It contributes to the initiation of the spike (S) protein of the coronavirus and, together with angiotensin-converting enzyme 2 (ACE2), assists the virus to enter target cells to infect the host (Hoffmann et al., 2020; He et al., 2021b).

Meanwhile, it is reported that TMPRSS2 was an important factor in cancer development (Hossain and Bostwick, 2013). TMPRSS2 has been demonstrated upregulated in prostate cancer cells by androgenic hormones and downregulated in prostate cancer tissue which is androgen-independent. The silence of TMPRSS2 could inhibit the proliferation of prostate cancer cells (Hossain and Bostwick, 2013). In breast cancer studies, the migratory and metastatic behavior of tumor cells can be promoted by the regulation of TMPRSS2 and its downstream signal pathway *in vitro* (Chi et al., 2020). In addition, studies have shown that patients with cancer are at greater risk of being infected by SARS-CoV-2 (Wang et al., 2021), considering the functions of TMPRSS2 in the COVID-19 infection, medicating the cancer patients infected COVID-19 requires thoughtful consideration.

Ginsenosides, as the main active components of ginseng, have a wide range of pharmacological effects (He et al., 2019), including antioxidant (He, 2017; Yang et al., 2018), antitumor (Zhou et al., 2018), anti-inflammatory (Chen et al., 2019; He and Li, 2015), anti-aging (Sun et al., 2018), etc., among which the antitumor effect is one of the hot spots of research. Many experiments have shown that ginsenosides can inhibit the invasion and metastasis of tumors (Wu et al., 2018). Studies have found that ginsenosides can inhibit the growth of liver cancer (Behrens et al., 2013). In addition, the extracts and metabolites of ginsenoside also have good inhibitory effects on lung cancer (Gao et al., 2013).

In this study, to gain a deeper understanding of how TMPRSS2 affects lung cancer, especially LUAD and LUSC, we used various types of bioinformatic approaches and IHC to determine the relationships between TMPRSS2 expression, methylation level, and OS, clinical parameters, biological process. Furthermore, we examined the correlation between TMPRSS2 and the level of TILs infiltration in tumor and normal tissue respectively. We tried to innovatively provide a novel insight for indicating the prognosis potential of LUAD patients from the perspective of immune infiltration and proposed a theoretical basis and novel therapeutic strategy for lung cancer treatment. In addition, we found that ginsenosides, as the inhibitors of TMPRSS2, play different roles in lung cancer and COVID-19 patients, providing new insights into the prevention and treatment of COVID-19 in lung cancer patients.

## 2 Materials and methods

### 2.1 Ethical approval

It was approved by the Independent Ethics Committee of Shanghai Jiao Tong University.

### 2.2 TMPRSS2 mRNA expression and the prognosis analysis

The mRNA expression level of TMPRSS2 in different types of cancer was identified by using TIMER 2.0 (Li et al., 2020) and UALCAN (Chandrashekar et al., 2017) database and the correlation between the expression of TMPRSS2 and the prognoses of lung cancer was examined by the PrognoScan (Mizuno et al., 2009), TIMER 2.0, GEPIA 2 (Tang et al., 2019) and Oncolnc (Anaya, 2016) database. The threshold was adjusted to a Cox *p*-value <0.05. The hazard ratio (HR) with 95% confidence intervals (CIs) and log-rank *p*-value were also determined.

In order to access the level of TMPRSS2 expression in different cell types of patients with lung cancer at single cell level, we utilized TISCH (Sun et al., 2021) to create an interactive gene expression visualization of multiple datasets at single-cell resolution across multiple datasets.

### 2.3 Clinical parameters analysis

The association between the mRNA expression level of TMPRSS2 and clinicopathological parameters was analyzed by UALCAN (a web tool for visualization of the impact between gene expression and clinicopathological parameters based on the TCGA data) and MEXPRESS (Koch et al., 2015) platform, which is used for integrating and visualizing clinical, expression and methylation data in TCGA at the single-gene level.

### 2.4 Correlation analysis

We determined the association between the mRNA expression level of TMPRSS2 and survival rate as well as different cancer staging in LUAD and LUSC (Lánczky and Győrffy, 2021). HRs with 95% CIs and log-rank *p*-values were also computed.

### 2.5 Methylation analysis

We employed UALCAN to examine the gene methylation levels across a range of clinicopathological features, such as ages and stages. The statistical significance was compared by using *t*-test. The MEXPRESS platform was also used to identify the level of methylation in the promoter region of TMPRSS2.

### 2.6 Biological network analysis and GSEA analysis

GeneMANIA (Warde-Farley et al., 2010) is used to create the biological network of TMPRSS2 based on the data relating to function associations, and LinkInterpreter modules in LinkedOmics (Vasaikar et al., 2018), which was used for enrichment analysis in TCGA, was employed for the identification of TMPRSS2 pathways and networks in LUAD and LUSC cohorts. Spearman’s correlation coefficient was used to investigate these results.

### 2.7 Relationship between immune infiltration levels and OS and its association with TMPRSS2 expression

TIMER2.0 was used to examine TMPRSS2 expression level in patients with lung cancer. The association between TMPRSS2 and the immune infiltrating level, including T cells (CD4^+^, CD8^+^), B cells, macrophages, neutrophils, and DCs was studied by using the gene modules in TIMER2.0 (Aran et al., 2015; Li et al., 2016). Log2 RSEM was used to determine gene expression levels. Then we used TIMER2.0 to analyze the relationship between the proportion of certain TILs and OS and the correlation with TMPRSS2 expression.

### 2.8 Correlation between TMPRSS2 expression and various immune cells in tumor and normal tissue

We employed GEPIA2 to evaluate the differences in the enrichment of different TILs including CD8^+^ T cells, Treg, macrophage, monocyte in the tumor and normal tissue of LUAD and LUSC patients, and further analyzed the enrichment of all T cell subtypes in the same patent cohorts (Danaher et al., 2017; Siemers et al., 2017).

### 2.9 Immunohistochemical staining for TMPRSS2 in lung cancer cohort tissue microarrays

Two lung cancer tissue microarrays were purchased from Superbiotek (Shanghai, PRC), containing 63 and 78 paired tumors and adjacent normal tissues from LUAD (LUC1601) and LUSC (LUC1602) patients, respectively. A summary of the clinical pathological information including subtype, tumor/nodal stage, histological grade, and information about patient follow-up. The tissue sections were stained with an anti-TMPRSS2 antibody (ab109131, ABCAM, United States) (dilution: 1: 1000). Following the general standard IHC staining methods, TMAs sections were used to measure the TMPRSS2 protein levels. The staining results were quantified according to the following criteria: for staining degree: 1, weak; 2, intermediate; 3, strong; For the proportion of positive cells: 1, 0–25%; 2, 26–50%; 3, 51–75%; 4, >75%. The staining score was obtained by multiplying staining degree by the proportion, 0–5 was considered low expression, and >5 was high expression.

### 2.10 Immunophenotyping of LUAD and LUSC patients

The Cancer Immunome Atlas (TCIA) database (Charoentong et al., 2017) contains immunogenomics characterizations of 20 solid cancers from TCGA, enabling comprehensive analysis of tumor immune and genetic profiles. Immunophenogram is used to classify patients who are likely to respond to antibody therapy targeting PD-1 by scoring a panel of immune genes. We analyzed the impact of TMPRSS2 expression on prognosis in the top 100 and bottom 100 patients by ranking the immunophenoscores (IPS) of LUAD and LUSC patients who were likely to respond to PD-1 antibody therapy and representative patients were selected to obtain the corresponding immunophenotype map.

### 2.11 Molecular docking

Docking calculations were performed by using the computerized protein-ligand docking software, Autodock Vina v.1.2.2 (Morris et al., 2008). TMPRSS2 was docked with the screened ginsenosides to obtain candidate ginsenosides that could be used as TMPRSS2 inhibitors.

### 2.12 Statistical analysis

HR and P or Cox *p* values based on a log-rank test. Spearman was used to assess the correlation. GraphPad Prism 7.0.was used for statistical analysis of results related to TMAs staining. Student’s *t* test was used to compare between 2 groups. Comparison among more than 2 groups was done using one-way analysis of variance (ANOVA). *p* values <0.05 were considered statistically significant.

## 3 Results

### 3.1 The variability of TMPRSS2 mRNA expression level across human cancer

**FIGURE 1:**
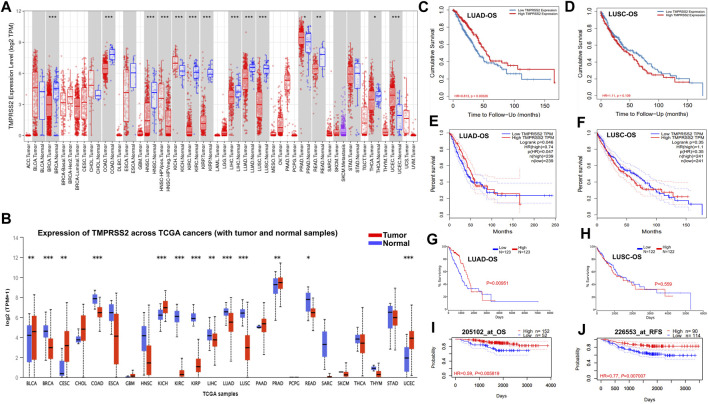
TMPRSS2 mRNA expression in different cancers and prognostic potential of TMPRSS2 in LUAD and LUSC.TMPRSS2 expression profile in different cancers *via* TIMER2.0 **(A)**. Blue and red dashed lines indicate the average value of normal and tumor tissues respectively. *p*-value Significant Codes: 0 ≤ *** <0.001 ≤ ** <0.01 ≤ * <0.05 ≤. < 0.1. The threshold was set as follows: *p*-value of 1E-6, fold change of 2, and gene ranking top 5%. TMPRSS2 expression profile in different cancers *via* UALCAN **(B)**. Blue and red dashed lines indicate normal and tumor tissues respectively; *p*-value Significant Codes: 0 ≤ *** <0.001 ≤ ** <0.01 ≤ * <0.05 ≤. < 0.1. Overall survival curves comparing the high and low expression of TMPRSS2 in LUAD **(C)** and LUSC **(D)** in the TIMER2.0. Overall survival curves comparing the high and low expression of TMPRSS2 in LUAD **(E)** and LUSC **(F)** in the GEPIA2. Overall survival curves comparing the high and low expression of TMPRSS2 in LUAD **(G)** and LUSC **(H)** in the Oncolnc database. OS **(I)** and RFS **(J)** comparing the high and low expression of TMPRSS2 in LUAD in the PrognoScan database.

Here, we found that TMPRSS2 expression was significantly lower in the tumor tissue of COAD, BRCA, HNSC, KIRC, KIRP, LIHC, LUAD, LUSC, READ, and THCA, whereas higher in KICH, PRAD, and UCEC compared with adjacent normal tissue (Figure 1A). The differential expression of TMPRSS2 between tumors and paired normal tissue for each of the TCGA tumors in UALCAN is displayed in Figure 1B. The expression of TMPRSS2 was significantly higher in CESC, BLCA, KICH, PRAD and UCEC, whereas lower in BRCA, COAD, KIRP, KIRC, LUAD, LUSC, LIHC, READ compared with adjacent normal tissues (Figure 1B). Integrating above results, we found that the mRNA expression level of TMPRSS2 in LUAD and LUSC tumor tissues is lower than that in the corresponding normal tissues.

### 3.2 The prognostic potential of TMPRSS2 in cancer

Notably, the expression of TMPRSS2 displays a significant association with OS in several types of cancers, including brain cancer, colorectal cancer, breast cancer, lung cancer, and ovarian cancer. The detailed association between TMPRSS2 mRNA expression and the prognostic potential of different cancers is presented in Supplementary Table S1.

In two lung cancer cohorts (GSE13213 and GSE4573), higher TMPRSS2 expression was associated to favorable outcomes (OS HR = 0.68, 95% CI = 0.51–0.89, Cox *p* = 0.006146; OS HR = 0.83, 95% CI = 0.70–0.0.99, Cox *p* = 0.042151) (Supplementary Table S1). Therefore, it is conceivable that a high level of TMPRSS2 may be independently linked to better prognoses of lung cancer. HR below 1 implies that TMPRSS2 expression has a protective effect on patients with lung cancer.

The LUAD cohort (GSE31210) demonstrated that a high level of TMPRSS2 was associated with improved OS (Figure 1I) and recurrence-free survival (RFS) (Figure 1J), but there is nonsignificant difference in LUSC cohorts. These data suggested that the expression level of TMPRSS2 shows different prognostic values based on cancer type. Moreover, TMPRSS2’s prognosis potential in LUAD and LUSC patient cohorts was also assessed by using the RNA-Seq dataset *via* TIMER 2.0, GEPIA2 and Oncolnc database, confirming the different association patterns between the level of TMPRSS2 and prognosis in LUAD and LUSC patient’s cohorts (Figures 1C–H).

### 3.3 Expression levels of TMPRSS2 impact the clinicopathological parameters in lung cancer

To better disclose the relevance and impact of the TMPRSS2 expression in LUAD and LUSC patients, the relationship between the mRNA expression level of TMPRSS2 and different clinical characteristics was studied (Supplementary Figure S1; Supplementary Table S2). We discovered that the expression level of TMPRSS2 was significantly different in each stage, different races, and different genders of both LUAD and LUSC patients (Supplementary Figures S1A–C, F–H). Moreover, the mRNA expression level of TMPRSS2 demonstrated age- and smoking-depended patterns in LUAD and LUSC cohorts (Supplementary Figures 1D, E, I, J; Supplementary Table S2). These phenomena indicate that the mRNA expression level of TMPRSS2 could impact the clinicopathological parameters of lung cancer but the differences in prognosis patterns between LUAD and LUSC are less affected by the TMPRSS2-depended clinicopathological parameters. So, there might be some other reasons lying behind.

For a greater understanding of the correlation and mechanism of TMPRSS2 expression level in LUAD and LUSC, we further studied the correlation between TMPRSS2 expression and clinical prognosis in patients with lung cancer with various clinicopathological factors. We found that a higher TMPRSS2 mRNA expression level was associated with a better OS for LUAD patients in the late stage, but worse OS for LUSC patients in the early stage (Table 1). Although both genders experienced better OS with higher TMPRSS2 expression in LUAD, male patients demonstrated better OS in both early and late stages compared with female patients. In addition, race-specific association patterns were observed in LUAD (Table 1). In addition to Asian races, TMPRSS2 has been linked to better OS in White races, and higher TMPRSS2 expression level in mutation burden LUAD was also associated with a better OS. All these results indicate that the expression level of TMPRSS2 has an impact on the prognosis of LUAD and LUSC with different patterns.

**TABLE 1:** Correlation of the mRNA expression level of TMPRSS2 in different stage and clinical prognostic potential in lung cancer with different clinicopathological factors.

Clinicopathological characteristics	Overall survival					
	LUAD (*n* = 513)			LUSC (*n* = 501)		
	N	Hazard ratio	*p*-value	N	Hazard ratio	*p*-value
Gender						
Female	270	0.58 (0.390.88)	**0.0086**	129	0.54 (0.25–1.16)	0.11
Male	234	0.55 (0.36–0.84)	**0.0051**	366	1.28 (0.94–1.75)	0.12
Race						
White	387	0.52 (0.37–0.73)	**0.00013**	348	1.28 (0.89–1.83)	0.18
Asian	---	---	---		---	---
Black/African American mutation burden	52	0.43 (0.12–1.52)	0.18	29	0.51 (0.15–1.79)	0.29
High	255	0.41 (0.22–0.76)	**0.0032**	240	0.84 (0.57–1.26)	0.41
Low	244	0.47 (0.31–0.72)	**0.00039**	242	0. (0.93–2.06)	0.11
Stage						
1	270	1.4 (0.84–2.33)	0.2	242	1.64 (1.05–2.55)	**0.027**
2	119	0.58 (0.32–1.06)	0.073	159	1.17 (0.7–1.95)	0.55
3	81	0.44 (0.24–0.8)	**0.0059**	83	0.54 (0.29–1.01)	0.05
4	26	0.14 (0.02–1.04)	**0.025**		---	---
Gender + Stage						
Early + male	66	0.45 (0.21–0.98)	**0.039**	119	1.23 (0.63–2.44)	0.54
Early + female	53	1.55 (0.51–4.69)	0.44	40	0.67 (0.21–2.12)	0.49
Late + male	37	0.38 (0.15–0.97)	**0.035**	63	0.55 (0.27–1.13)	0.098
Late + female	44	0.44 (0.016–1.19)	0.098	20	0.31 (0.08–1.21)	0.075

Bold values indicate *p* < 0.05.

### 3.4 Protein level of TMPRSS2 impacts the clinicopathological parameters of LUAD

**FIGURE 2:**
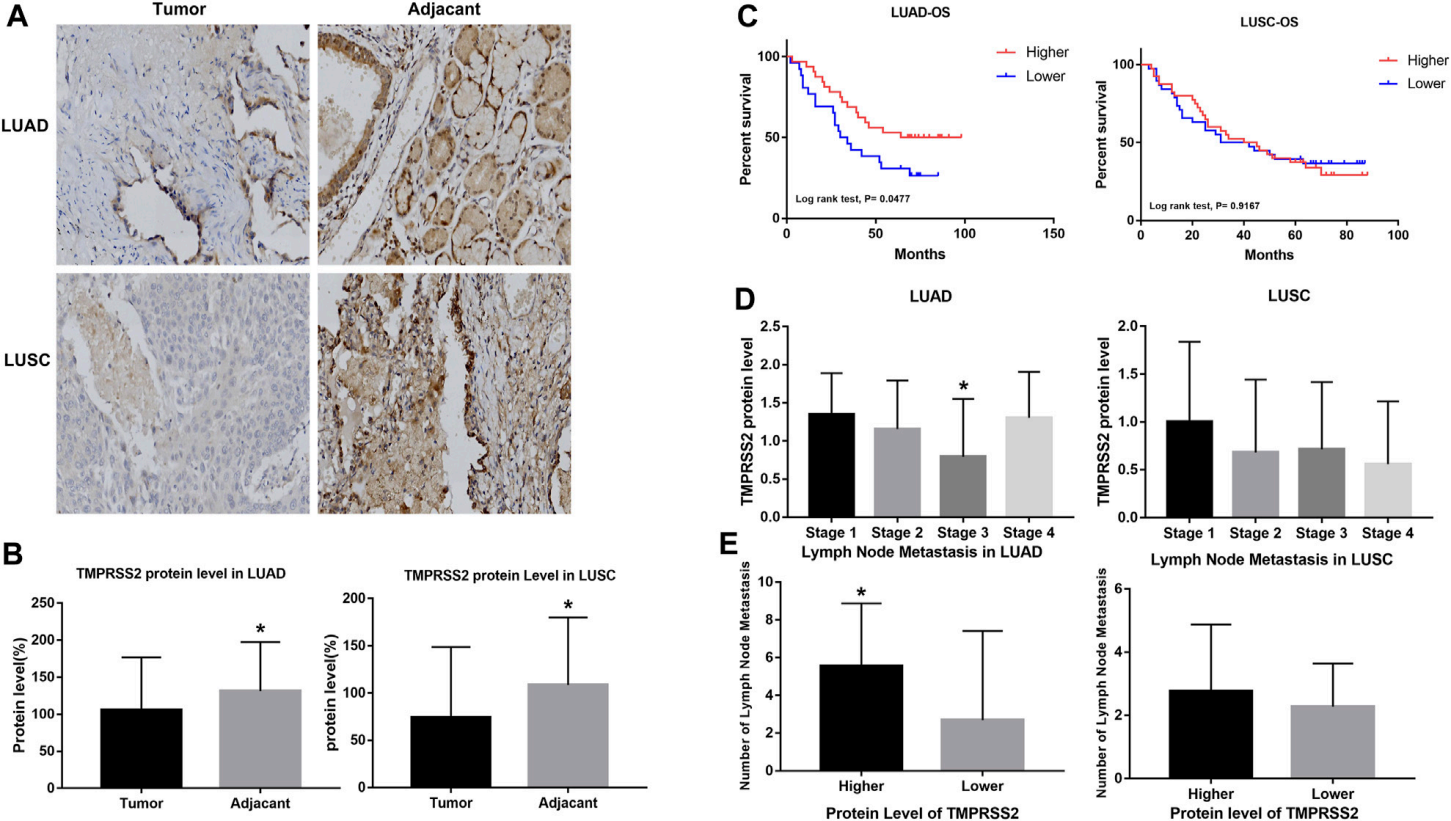
Higher expression of TMPRSS2 protein was correlated with different clinicopathological parameters in LUAD and LUSC cohorts.**(A)** Immunohistochemical staining for TMPRSS2 of tumor and adjacent tissue in LUAD and LUSC. Original magnification ×40. TMPRSS2 protein level in tumor and adjacent **(B)** and overall survival **(C)** of LUAD and LUSC, *p < 0.05. **(D)** Correlation between TMPRSS2 protein level and clinical stage, *p < 0.05. **(E)** Correlation between TMPRSS2 protein level and lymph node metastasis, *p < 0.05.

We examined TMPRSS2 protein levels in two tissue microarrays containing 63 and 78 paired tumor and normal tissues, respectively, from LUAD and LUSC patient cohorts. Both of those have detailed clinicopathological information, including histological grading, subtype, tumor/nodal stage, and patients’ follow-up over 5 years information. TMPRSS2 is mainly expressed in the cytoplasm (Figure 2A). The protein level of the tumor was significantly lower compared to the paired normal tissue in both LUAD and LUSC patient cohorts (Figure 2B). Next, we examined the relationship between TMPRSS2 protein level and the clinical parameters. We found that a higher protein level of TMPRSS2 was associated with a better OS in LUAD patient cohorts rather than in LUSC cohorts (Figure 2C). Moreover, we also found a negative correlation between lower TMPRSS2 levels and clinical stages, and lymph node metastasis status in LUAD patients (Figures 2D, E).

To validate the reliability of our findings, we also analyzed the correlation between TMPRSS2 protein levels in LUAD cohorts and different clinical parameters *via* UALCAN (Supplementary Table S3). We found a significant decrease in TMPRSS2 protein level in LUAD compared to normal tissues, with the most significant decrease in solid adenocarcinoma (Supplementary Figures 2A, H). Furthermore, we also found that TMPRSS2 protein was significantly reduced in male LUAD patients and correlated with higher tumor grade (Supplementary Figures 2D, G). In the pathway-related studies, we found that the protein level of TMPRSS2 was significantly associated to HIPPO pathway status, mTOR pathway status, WNT pathway status, NRF2 pathway status, P53/Rb-related pathway status, RTK pathway status, SWI-SNF complex status and chromatin modifier status (Supplementary Figures S2I–P), indicating that TMPRSS2 may function through these pathways in LUAD.

### 3.5 Low promoter methylation level of TMPRSS2 impacts the clinicopathological parameters of patients with lung cancer

**FIGURE 3:**
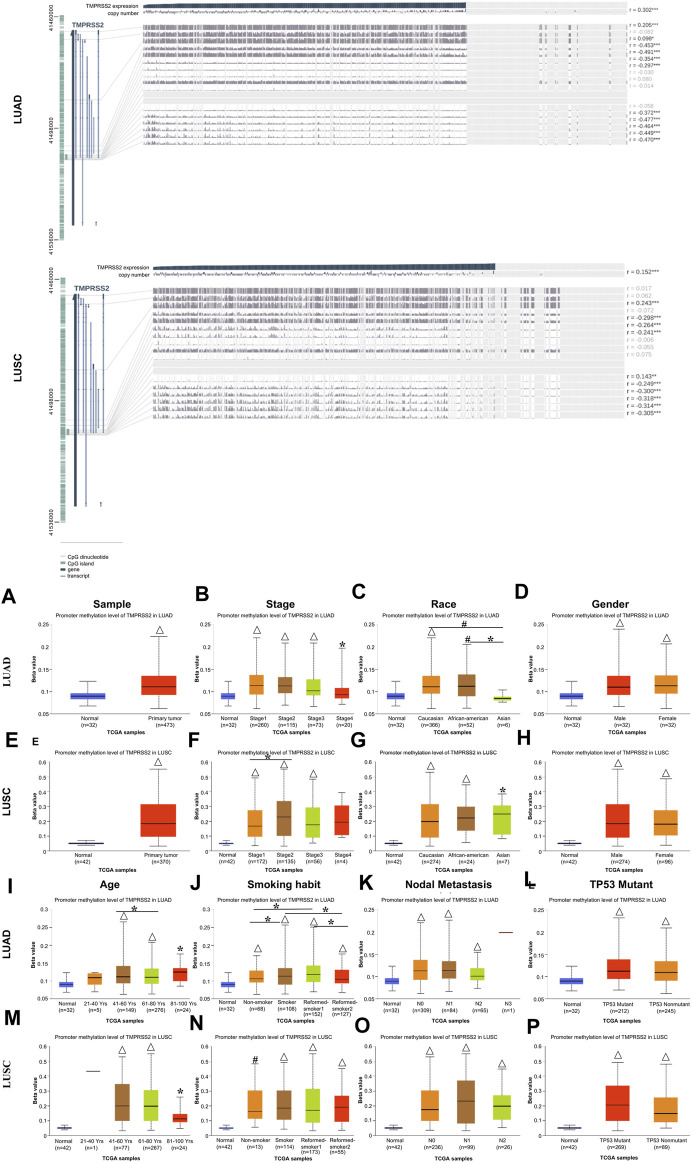
Visualization of promoter methylation levels of TMPRSS2 impact the clinicopathological parameters in LUAD and LUSC using MEXPRESS (upper) and UALCAN (lower).The promoter methylation level of TMPRSS2 of sample types **(A, E)**, stage **(B, F)**, race **(C, G)**, gender **(D, H)**, age **(I, M)**, smoking habit **(J, N)**, nodal metastasis status **(K, O)** and TP53 mutant **(L, P)** in LUAD and LUSC cohorts respectively. *p ≤ 0.05, #*p* ≤ 0.001, △*p* ≤ 0.0001.

To reveal the mechanism involved in the decreased TMPRSS2 expression in LUAD and LUSC, we predicted the methylation site and examined the methylation of TMPRSS2 in tumors and normal tissues of LUAD and LUSC respectively *via* MEXPRESS and UALCAN (Figure 3; Supplementary Tables S4, S5).

Among these CpG locations, CpG41504034 drew our attention, since TMPRSS2 expression level is significantly negatively correlated with the methylation status in a LUAD cohort, whereas the level of TMPRSS2 is significantly positively correlated with the methylation status in CpG 41508181 in LUSC patients (Supplementary Table S4). Moreover, the methylation status in the LUAD patients is significantly lower than that of LUSC on the CpG 41488290, while significantly positively correlated in the LUSC cohort.

We found that 5 CpG sites had significantly higher hypermethylation levels in the tumor tissue compared to paired normal tissue of LUAD and LUSC patients (*p* < 0.0001, Figures 3A, E). A higher level of promoter methylation was detected in the early stage, suggesting that the high levels of TMPRSS2 promoter methylation were related to earlier stages of lung cancer development (Figures 3B, F). According to the methylation analysis here, the level of TMPRSS2 may fluctuate throughout the course of lung cancer. Additionally, a similar pattern was observed in the analysis of nodal metastasis, suggesting that the levels of TMPRSS2 promoter methylation are somehow related to nodal metastasis (Figures 3K, O). Race and age-related methylation patterns of TMPRSS2 promoters have similar patterns in LUAD and LUSC, respectively; that is, the African-American group, as well as the younger group of these two cohorts, had higher methylation levels of TMPRSS2 (Figures 3C, G, I, M). In addition, TMPRSS2 methylation level was also associated with gender, smoking habits and TP53 mutants (Figures 3D, H, J, N, L, P).

The effect of methylation level on prognosis is consistent with the previously found effect of mRNA expression level on prognosis. That is, the level of TMPRSS2 expression and methylation are basically the same in the two lung cancer subtypes, so we believe that the difference between TMPRSS2 on LUAD and LUSC prognosis was also not at the methylation level.

### 3.6 Gene ontology of TMPRSS2

**FIGURE 4:**
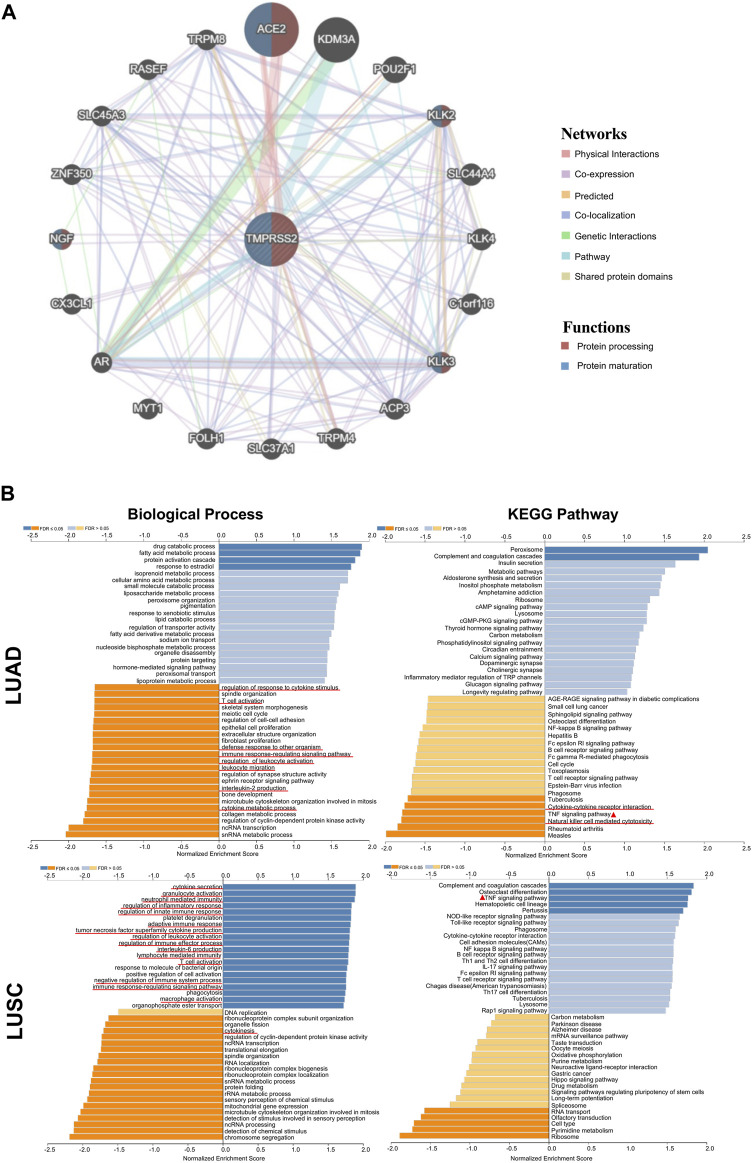
TMPRSS2-related gene enrichment analysis by using GENEMANIA and LinkedOmics. **(A)** Interaction network of TMPRSS2 in TCGA. Lines with different colors represent different bioinformatics methods and different colors in the ring represent diverse functions of the genes. **(B)** Enriched gene ontology annotations of biological process and KEGG pathway analysis of TMPRSS2-related genes in LUAD and LUSC. Red signifies positive and blue signifies negative. Light blue and orange signify FDR >0.05 while dark blue and orange represent FDR ≤0.05.

In order to investigate the mechanism of TMPRSS2 in biological processes, we performed the GSEA analysis of its related genes. Figure 4A shows 20 TMPRSS2-related proteins based on the analysis of physical interactions, co-expression, co-localization, predicted, genetic interactions, pathway and shared protein domain that was screened through the GENEMANIA database. We found that TMPRSS2 has a high correlation with ACE, KLK2, KLK3, and NGF in protein processing and maturation. In addition, ACE, KLK2, KLK3, and NGF as a gene set positively correlated with prognosis in LUAD but not LUSC (Supplementary Figure S3), which reconfirmed our previous finding that the mRNA expression of TMPRSS2 has different correlation patterns with the prognosis of LUAD and LUSC, that is, the higher the expression level, the better the prognosis.

Furthermore, we used LinkedOmics database to further analyze TMPRSS2-related genes in LUAD and LUSC cohorts. The pathways and networks were identified using LinkInterpreter.

We found that TMPRSS2 functions in LUAD to downregulate regulation of response to cytokine stimulus, T cell activation, defense response to other organism, immune response-regulating signaling pathway, regulation of leukocyte activation, leukocyte migration, interleukin-2 production and cytokine metabolic process (Figure 4B), However, immune-related biological processes were mostly up-regulated in LUSC patients, suggesting that TMPRSS2 is closely related to immune biology in LUAD and LUSC, and likely in opposite patterns. Further, we found that the relationship between TMPRSS2 and TNF signaling pathway in KEGG pathway analysis showed two diametrically opposite patterns in LUAD and LUSC (Figure 4B). This suggests that: ① the underlying mechanism of the different correlation patterns between the TMPRSS2 mRNA expression level and the prognosis might be the immunity regulatory; ② TMPRSS2 has opposite biological effects on TNF activation.

### 3.7 TMPRSS2 expression is correlated with immune infiltration level in lung cancer

**FIGURE 5:**
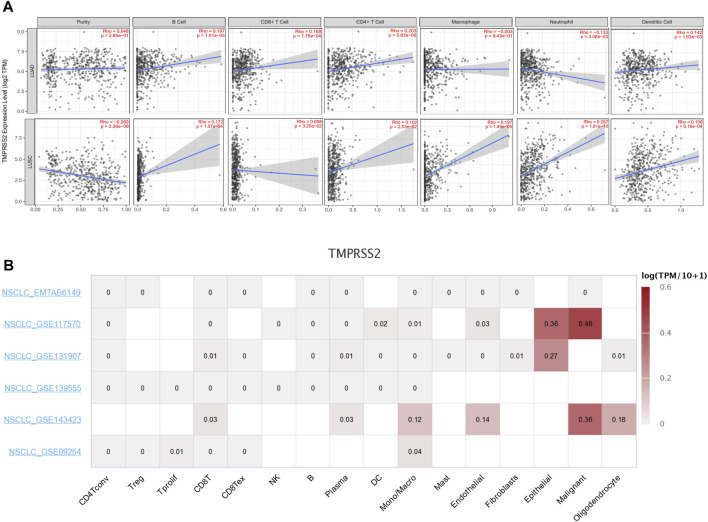
Correlation between TMPRSS2 expression and immune infiltration level in LUAD and LUSC by using TIMER 2.0.Including B cell, CD8^+^ T cells, CD4^+^ T cells, macrophages, neutrophils, and dendritic cell **(A)**. Average expression of TMPRSS2 in different cell-types across NSCLC datasets by using TISCH. The color indicates gene expression level **(B)**.

Tumor-infiltrating lymphocytes are independent predictors of survival in cancers. Therefore, we investigated whether the expression of TMPRSS2 was correlated with immune infiltration levels in lung cancer. We assessed the correlations between TMPRSS2 expression and immune infiltration levels in LUAD and LUSC from TIMER2.0. A significant negative correlation was found between TMPRSS2 expression and tumor purity in LUSC, which suggests that TMPRSS2 may be linked to lymphocyte recruitment to LUSC niches and significant positive correlated to macrophages and neutrophils infiltration levels in LUSC (Figure 5A), while macrophages and neutrophils infiltration were negative correlated to TMPRSS2 expression level in LUAD. In addition, the expression level of TMPRSS2 demonstrated significant positive correlations with infiltrating levels of B cells, CD8^+^ T cells, CD4^+^ T cells, and dendritic cells in both LUAD and LUSC (Figure 5A).

In order to clarify whether the correlation between TMPRSS2 and immune infiltration is due to the recruitment of immune cells by TMPRSS2, or whether immune cells themselves express TMPRSS2, we used single-cell sequencing data from patients with NSCLC in the TSICH database to study the expression of TMPRSS2 in different cells (Figure 5B) and found that TMPRSS2 was mainly expressed in epithelial cells and malignant cells, but was hardly expressed in immune cells. It indicated that the relationship between the level of TMPRSS2 expression and immune infiltration was correlated with immune cells recruitment by TMPRSS2.

**FIGURE 6:**
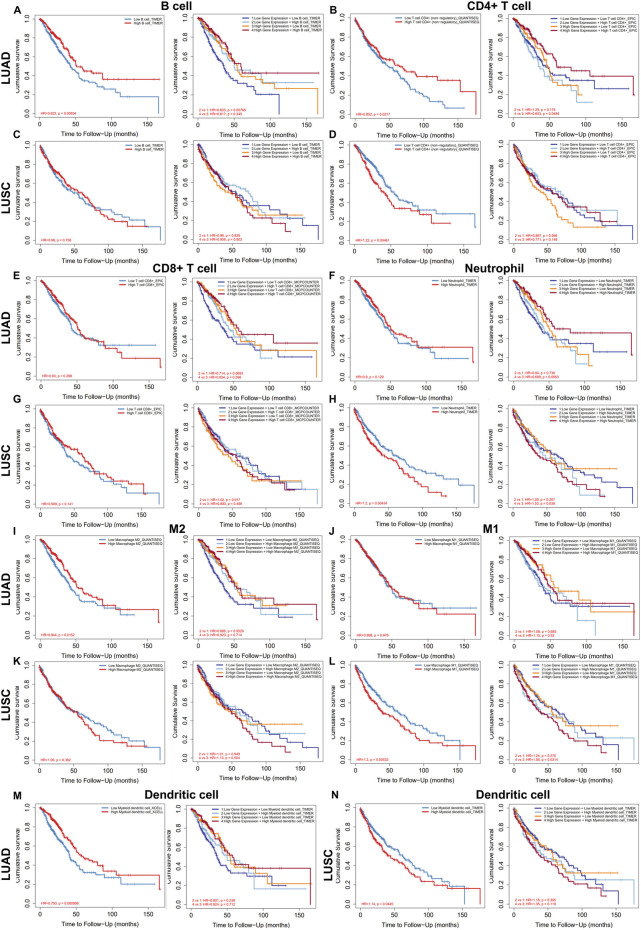
Relationship between the level of immune infiltration and OS and its synergy with TMPRSS2 expression in LUAD and LUSC by using TIMER 2.0. Including B cell **(A,C)**, CD4^+^ T cells **(B,D)**, CD8^+^ T cells **(E,G)**, neutrophils **(F,H)**, M2 **(I,K)**, M1 **(J,L)** and dendritic cell **(M,N)**.

We further explored the prognostic impact of TMPRSS2 expression and immune infiltration in LUAD and LUSC, we studied the relationship between different immune cells and lung cancer OS, and the relationship between the combined effect of TMPRSS2 and immune cells and lung cancer OS respectively (Figure 6). We found that although TMPRSS2 and B cells, CD4^+^T cells are positively correlated in both LUAD and LUSC, the proportion of B cells and CD4^+^T cells and prognosis have different patterns in the two subtypes (Figures 6A–D), that is, the higher the percentage of B cells, the better the prognosis of LUAD, but not LUSC (Figures 6A, C). As for CD4+T cells, the higher the percentage of CD4^+^T cells, the worse the prognosis of LUSC (Figure 6D), so the positive correlation between TMPRSS2 expression and CD4^+^T cells will augment the poor prognosis of LUSC; similarly, the higher the proportion of neutrophil, the worse the prognosis of LUSC (Figure 6H), so the positive correlation between TMPRSS2 expression and neutrophil in LUSC will also lead to poor prognosis of LUSC.

We also found that different subtypes of macrophages play different roles in LUAD and LUSC (Figures 6I–L). The high expression of TMPRSS2 mRNA in LUSC is positively correlated with macrophages, which may be one of the reasons why TMPRSS2 is not a favorable factor for LUSC.

In addition, the infiltration of DCs and the effect of TMPRSS2 expression on prognosis were also diametrically opposite between LUAD and LUSC (Figures 6M, N), which suggested that the underlying mechanism of TMPRSS2 leading to a different prognosis pattern in two lung cancer subtypes might be the recruitment and infiltration of TILs.

Furthermore, we explored the potential prognostic significance of infiltrating cells of T-cell (Tregs, T cell follicular helper, T cell CD4^+^ memory resting and T cell NK) in LUAD and LUSC (Supplementary Figure S4). We found that the infiltration of all these 4 types of T cells correlated to a better prognosis in LUAD rather than in LUSC (Supplementary Figures S4A–H). Moreover, besides Tregs and T cell CD4^+^ memory resting, the expression level of TMPRSS2 augments the positive association between T cell NK and LUAD prognosis (Supplementary Figure S4F). Also, there is a difference in the correlation pattern between the infiltration level of Treg and prognosis in LUAD and LUSC patients (Supplementary Figures S4A, C). It is shown that the higher infiltration level of Treg, the better the prognosis of LUAD whereas the poorer prognosis of LUSC, which implies that the positive correlation between TMPRSS2 and Treg will worsen the poor prognosis of LUSC. These results further illustrate that the underlying mechanism by which TMPRSS2 leads to different prognostic patterns in LUAD and LUSC may be the recruitment and infiltration of TILs. Therefore, we will study the TILs in the tumor and normal tissue of LUAD and LUSC respectively.

### 3.8 Correlation analysis between TMPRSS2 expression and immune cells in the tumor and normal tissue of lung cancer

**FIGURE 7:**
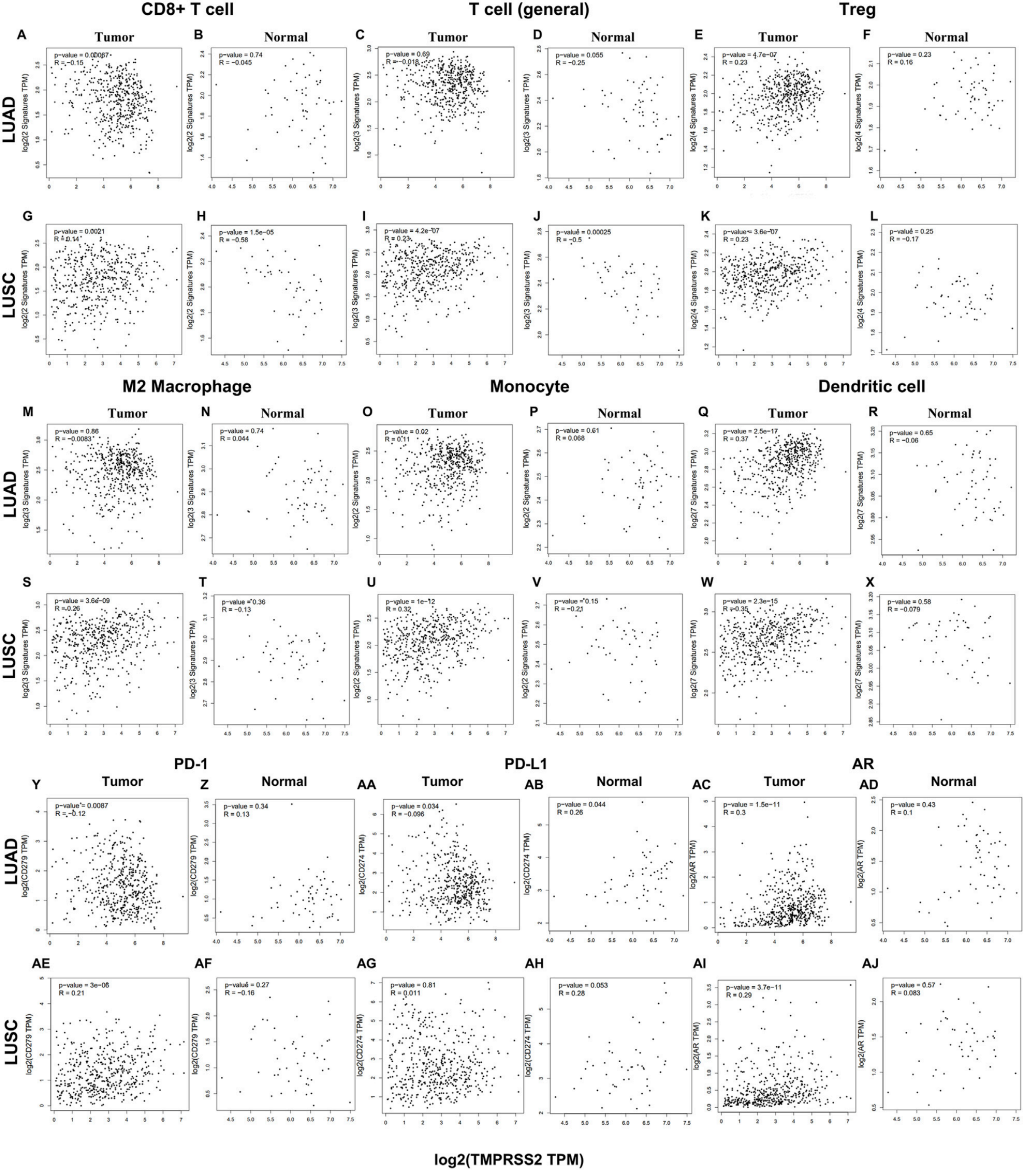
Correlation between TMPRSS2 expression and different immune cells in tumor and normal tissue of LUAD and LUSC by using GEPIA2. Markers include general T cell (CD2, CD3D, CD3E); CD8^+^ T cell (CD8A, CD8B); exhaust-ed T cells (CTLA4, PDCD1, LAG3, GZMB and HAVCR2); Treg (CCR8, FOXP3, TGFB1, and STAT5B); monocytes (CD86, CSF1R); M2 macrophages (VSIG4, MS4A4A, and CD163); Dendritic cell (CD1C, HLA-DPA1, HLA-DPB1, HLA-DQB1, HLA-DRA, ITGAX, and NRP1). Scatterplots of correlations between TMPRSS2 expression and gene markers of CD8^+^ T cell **(A, B)**, T cell (general) **(C, D)**, Treg **(E, F)**, M2 Macrophage **(M, N)**, Monocyte **(O, P)**, Dendritic cell **(Q, R)**, PD-1 **(Y, Z)**, PD-L1 **(AA, AB)**, AR **(AC, AD)** in tumor and normal tissue of LUAD; Scatterplots of correlations between TMPRSS2 expression and gene markers of CD8^+^ T cell **(G, H)**, T cell (general) **(I, J)**, Treg **(K, L)**, M2 Macrophage **(S, T)**, Monocyte **(U, V)**, Dendritic cell **(W, X)**, PD-1 **(AE, AF)**, PD-L1 **(AG, AH)**, AR **(AI, AJ)** in tumor and normal tissue of LUSC.

To evaluate the relationship between TMPRSS2 and immune infiltrating cells, we focused on the correlations between TMPRSS2 and immune cells, including general T cells, CD8^+^ T cells, Treg, M2 macrophages, monocyte, and DC in tumor and normal tissue of lung cancer. In addition, we also examined the correlation between the expression level of TMPRSS2 and the level of PD-1, PD-L1, and AR (Figure 7). After adjusting by purity, the results demonstrated a significant negative correlation between TMPRSS2 expression level and CD8^+^T cells and T cells (general) in the normal tissue of LUSC (Figures 7H, J). Moreover, no significant correlation was detected in the normal tissue of M2 macrophages and monocyte in lung cancer cohorts (Figures 7N, P, T, V). The thing that interested us was that different correlation patterns were demonstrated in CD8^+^ T cells and general T cells in tumor and normal tissue in LUAD and LUSC. In the tumor tissue, TMPRSS2 was negatively correlated with CD8^+^ T cells in LUAD cohort but positively correlated in LUSC cohort (Figures 7A, G). General T cells were positively correlated with TMPRSS2 in LUSC cohort but have no correlation with TMPRSS2 in the tumor tissue of LUAD patients (Figures 7C, I).

M2 macrophages are tumor-promoting cells and TMPRSS2 was significantly associated with M2 in the tumor tissue of LUSC rather than in LUAD (Figures 7M, S).

The association between TMPRSS2 and Treg, monocyte and DC is consistent in LUAD and LUSC tumor tissues (Figures 7E, K, O, U, Q, W). In addition, TMPRSS2 was negatively correlated with CD8^+^ T cells and T cells (general) in LUSC normal tissues, but not significant negatively correlated in LUAD normal tissues (Figures 7B, D, H, J).

We also found that TMPRSS2 was positively associated with PD-1, and AR in LUAD normal tissues but the difference was not significant (Figures 7Z, AD). Moreover, the expression level of TMPRSS2 and PD-1 and PD-L1 demonstrated different correlation patterns in the tumor tissue of lung cancer (Figures 7Y, AA, AE, AG). Also, we found that TMPRSS2 was positively related to AR and negatively associated with PD-1 and PD-L1 in LUAD tumor tissue (Figures 7Y, AA, AC), suggesting that TMPRSS2 agonists might be an immunomodulator of LUAD to anti-PD-1 based immunotherapy combination therapies.

So, all these above are indicated that the different prognosis patterns might be induced by the different immune infiltrating patterns. Further research is needed to determine whether TMPRSS2 is crucial in mediating immune cells recruitment and remodeling the tumor microenvironment.

Also, we examined different functional T cells, such as Th1 cells, Th2 cells, Th17 cells, Tfh cells, and Tregs, as well as exhausted T cells, which are all essential elements involved in tumor immune infiltrating. It was found that TMPRSS2 was positively related to naive T-cells, resident memory T-cells, central memory T-cells, exhausted T cells, resting Treg, and Th1-like cells in LUAD normal tissues but the difference was not significant (Supplementary Figures 5B, N, P, R, Z, AD). Moreover, the expression level of TMPRSS2 and the effector memory T-cell, resting Treg, T cell exhaustion, and effector Treg demonstrated different correlation patterns in the tumor tissue of lung cancer (Supplementary Figures 5E, K, Q, W, Y, AE, AA, AG). These might be the reason that higher TMPRSS2 expression levels leading a better prognosis in LUAD patients.

### 3.9 Prediction of the relationship between TMPRSS2 expression and response to PD-1 blockade immunotherapy in patients with lung cancer

**FIGURE 8:**
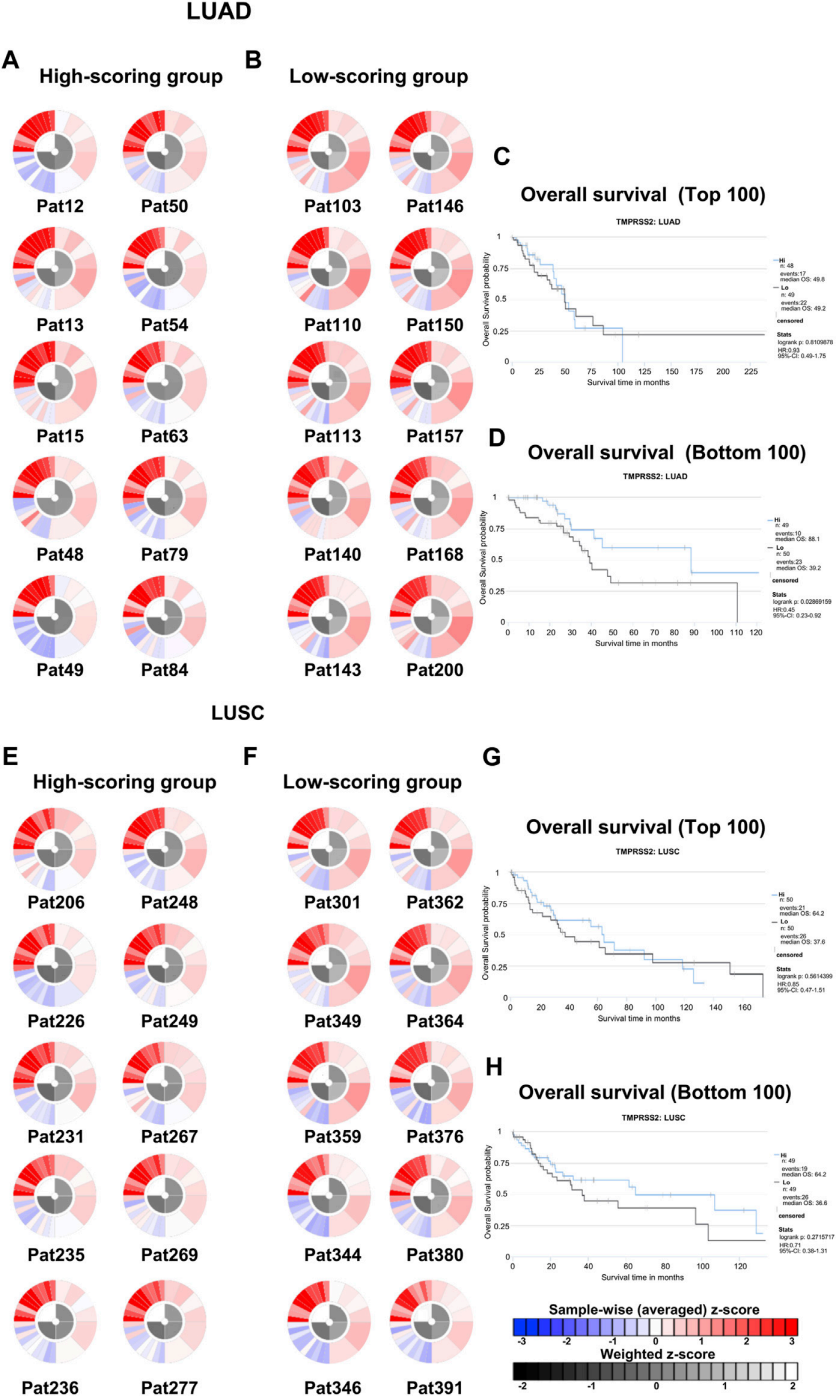
Immunophenoscores and overall survival of patients with lung cancer that response to PD-1 blockers immunotherapy in TCIA. **(A)** Immunophenograms for individual patients in the top 100 IPS_PD-1_pos patients with LUAD, The wheel contains individual factors were classified into four categories (clockwise direction), effector cells (EC), suppressor cells (SC), immunomodulators (CP), and MHC molecules (MHC). Effector cells including activated CD4^+^ T cells, activated CD8^+^ T cells, effector memory CD4^+^ T cells, effector memory CD8^+^ T cells. Immunosuppressive cells including MDSC and Treg. Immune checkpoint markers include LAG3, PDCD1, CTLA4, TIGIT, PDCD1LG2, HAVCR2, CD274, IDO1, CD27, ICOS, and B2M. MHC molecules including TAP1, TAP2, HLA-A, HLA-B, HLA-C, HLA-DPA1, HLA-DPB1, HLA-E, and HLA-F in the clockwise direction. The data displayed by the weighted average z-scores for the elements within the particular category (z > 0; showed in red, z < 0; showed in blue) **(B)** Immunophenograms for individual patients in the bottom 100 IPS_PD-1_pos patients with LUAD. **(C)** Overall survival of the top 100 IPS_PD-1_pos patients with LUAD. **(D)** Overall survival of the bottom 100 IPS_PD-1_pos patients with LUAD. **(E)** Immunophenograms for individual patients in the top 100 IPS_PD-1_pos patients with LUSC. **(F)** Immunophenograms for individual patients in the bottom 100 IPS_PD-1_pos patients with LUSC. **(G)** Overall survival of the top 100 IPS_PD-1_pos patients with LUSC. **(D)** Overall survival of the bottom 100 IPS_PD-1_pos patients with LUSC.

Due to intrinsic immune resistance, only a minority of cancer patients benefit from anti-PD-1 therapy. We used the TCIA database to investigate whether TMPRSS2 influences the response of patients with lung cancer to anti-PD-1 immunotherapy. We obtained and ranked IPS that predict response to PD-1 blockers in patients with LUAD and LUSC from the TCIA database (Figure 8; Supplementary Table S6). The top 100 and bottom 100 patients were selected for follow-up analysis. Immunophenograms of representative patients were obtained from the top 100 and bottom 100 patients of LUAD and LUSC cohorts respectively (Figures 8A, B, E, F). We found that high-scoring patients and low-scoring patients had different expression patterns. Immunosuppressive cells were enriched in tumors of low-scoring patients compared with the high ones. In addition, we studied the association between the expression of TMPRSS2 and prognosis in high- and low-scoring patients, respectively (Figures 8C, D, G, H). And found that the mRNA expression of TMPRSS2 in LUAD was significantly positive associated with prognosis in low-scoring patients. All these above imply that TMPRSS2 might be an immunomodulator in patients who have no response to anti-PD-1 therapy.

### 3.10 Screening of candidate TMPRSS2 inhibitors

We obtained a collection of ginsenosides that could serve as TMPRSS2 inhibitors by screening. To evaluate the affinity of the drug candidates for their targets, we performed molecular docking analysis and five ginsenosides that could bind to TMPRSS2 were obtained, namely ginsenoside F1, ginsenoside Rh1, ginsenoside C-K, ginsenoside Rk3 and (20R)-Ginsenoside Rh1, and yielded binding energies per interaction (Table 2). We found that the binding energies of these five ginsenosides to TMPRSS2 were all greater than 6 kcal/mol, indicating that they have good binding activity to TMPRSS2.

**TABLE 2:** Five candidate ginsenosides as TMPRSS2 inhibitors.

Compounds	CAS	Formula	MW	Binding energy (kcal/mol)	Scores (kcal/mol)	Ki (uM)	Structure
Ginsenoside F1	53963-43-2	C8H1867ClN	638.88	−6.573	−9.6	0.090228	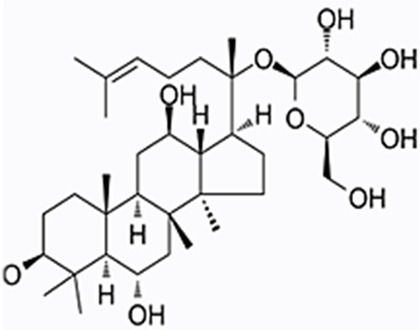
Ginsenoside Rh1	63223-86-9	C8H2145ClN	638.87	−6.211	−8.6	0.488831	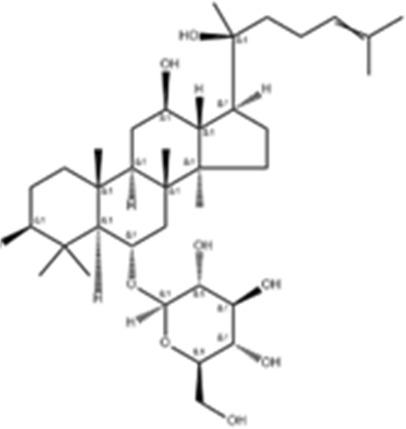
Ginsenoside C-K	39262-14-1	C8H1752ClN	622.88	−6.593	−8.5	0.578816	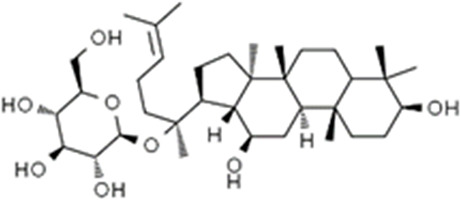
Ginsenoside Rk3	364779-15-7	C8H659ClN	620.86	−6.978	−8	1.347256	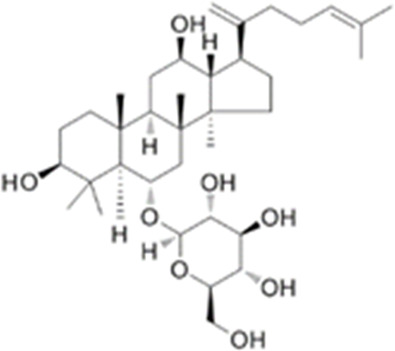
(20R)-Ginsenoside Rh1	80952-71-2	C8H1769ClN	638.9	−7.108	−6.8	10.2337	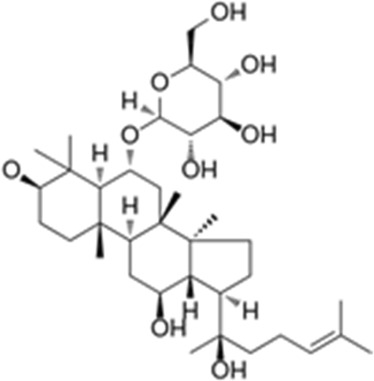

## Discussion

As a type II transmembrane serine endopeptidase, TMPRSS2 is conserved in many organisms (Shen et al., 2017). It is expressed in many tissues, such as lung, liver and prostate, etc., among which it is mainly expressed in the prostate in an androgen-dependent manner (Lin et al., 1999). TMPRSS2 plays a role in many pathophysiological processes, including digestion, tissue remodeling, inflammation, tumor cell infiltration, apoptosis, and pain (Lam et al., 2015; Thunders and Delahunt, 2020). Emerging researches have demonstrated a functional association between TMPRSS2 and various types of disease, especially tumors (Hong et al., 2020). Whether TMPRSS2 is involved in various tumors’ pathogenesis *via* certain molecular mechanisms remains to be elucidated. TMPRSS2 has been found to inhibit colorectal cancer cell migration (Bowden et al., 2007), and its expression was diminished in cases with P53 mutations (Nishijima et al., 2015). In addition, TMPRSS2 was also taken a key part in HCC by driving the recruitment and differentiation of peritumoral fibroblasts into TAMs (Thunders and Delahunt, 2020). These findings suggest the involvement of TMPRSS2 in TME. Immunotherapy, the new treatment method available to patients, is associated strongly and consistently with TME.

In this study, we comprehensively studied the mRNA expression, protein level, molecular features of gene expression, DNA methylation, genetic alteration, and protein level of TMPRSS2 in two subtypes of NSCLC relying on various types of computational approaches. Moreover, this finding has been verified by IHC staining study.

According to our study, variations in TMPRSS2 expression level influence prognosis across various cancers, and a higher level of TMPRSS2 associated with better prognosis in LUAD cohorts. Furthermore, our analyses based on databases show that prognosis patterns among lung cancer subtypes differ according to immune infiltration levels. Consequently, our research also offers novel insights into TMPRSS2’s possible role in tumor immunology as well as its potential use as a biomarker and novel therapeutic target for lung cancer.

Studies have shown that androgens can promote the exhaustion of CD8^+^T cells by regulating the transcription factor Tcf7, thereby promoting tumor growth. Blockade of androgen receptors can significantly enhance the efficacy of anti-PD-1 therapy (Thunders and Delahunt, 2020). We found that in LUAD, compared with normal tissues, AR was significantly positively correlated with TMPRSS2 in tumor tissues, this was also confirmed in the protein expression levels of TMPRSS2 in different sexes (Figure 7AC; Supplementary Figure S2D). Additionally, we also found a significant decrease of TMPRSS2 in tumor tissues of patients with lung cancer. Also, a significant negative correlation was observed between TMPRSS2 and PD-1 and PD-L1 expression. Furthermore, the correlation between TMPRSS2 and the prognosis of patient who have no response to anti-PD-1 immunotherapy was positive, suggesting that agonists of TMPRSS2 may benefit the treatment of LUAD patients who are not suitable for PD-1 inhibitor therapy. These above suggest that: ① TMPRSS2 might be a new target for combined therapies based on anti-PD-1; ②This combined strategy may significantly extend cancer immunotherapy and improve the clinical benefits of male LUAD patients; ③ Among these, the advanced patients may profit more from the therapeutics above. These conclusions are based on sequencing data from a large database using bioinformatics approaches and IHC study on over one hundred paired tumors and adjacent normal tissues from LUAD and LUSC patients, respectively. In the future, we plan to carry out wet-bench experiments, such as flow cytometry analysis to determine the ratio of different types of immune cells in lung cancer mice model to minimize these limitations mentioned above and to make our conclusions as solid as possible. Ginsenosides, as the main active substances in ginseng, have a wide range of antitumor activities. After relevant screening and molecular docking analysis, five ginsenosides that could bind to TMPRSS2 were finally identified. The selectivity of TMPRSS2 inhibitors will be further evaluated to identify small molecules with higher selectivity for TMPRSS2. In addition, ginsenosides will play a role in the prevention and treatment of COVID-19 as an inhibitor of TMPRSS2. However, as TMPRSS2 inhibition is detrimental to the prognosis of LUAD patients who have no response to anti-PD-1 therapy, its use in the prevention and treatment of COVID-19 in LUAD patient needs to be thoughtfully considered, since the different roles of TMPRSS2 expression level in patients with COVID-19 infection and in LUAD patients who do not respond to PD-1 treatment, trade-offs need to be made in medicating TMPRSS2 inhibitors, such as ginsenosides to gain prophylactic and therapeutic benefits against COVID-19 and treatment in LUAD patients. The five ginsenoside candidates we selected here are based on virtual screening, so we believe that further pharmacological experiments are necessary to verify our findings. We started to administer these five ginsenoside compounds to LUAD and LUSC mice model to observe the tumor biology and the recruitment of immune cells *in vivo*, which will facilitate to improve the credibility of our findings.

This study provides an overview of TMPRSS2 expression and lung cancer. All these correlations above suggest the potential mechanism where TMPRSS2 regulates T cell functions in LUAD. All these findings suggest that TMPRSS2 takes a vital part in the recruitment and regulation of NK cells and effective T cells, leading to a better prognosis.

Toll-like receptors (TLRs), a type I membrane protein, play a role in recognizing various pathogen-associated molecular patterns (PAMPs) to initiate innate immunity, and are very important for early host defense (Owen et al., 2020). In addition, TLRs maintain tissue homeostasis and promote antitumor effects through activation and modulation of adaptive immune responses. As potent immune stimulators, the binding of TLR agonists to TLRs promotes the maturation of antigen-presenting cells, activates downstream signaling pathways, such as MAP kinases, NFκB, and IRF, and disrupts immunosuppression and tolerance, thereby enhancing innate or treatment-induced antitumor immune response (Owen et al., 2020; Kayesh et al., 2021). A study on primary human nasal epithelial cells found that the TLR3 agonist Poly (I: C) can activate IFN and NFκB signaling pathways after binding to TLR3, thereby increasing the expression levels of ACE2 and TMPRSS2 (Nakazono et al., 2021). Since the TLR agonist poly (I: C) can enhance the expression of TMPRSS2 and act on innate immune cells, this means that the use of TLR agonists in lung cancer will enhance the efficacy of PD-1 targeting adaptive immune cells (Nakazono et al., 2021). Moreover, the researchers found that cancer patients were more vulnerable to SARS-CoV-2 infection and, thus, had worse clinical outcomes (Dai et al., 2020). So this suggests that we should pay more attention to cancer patients in the COVID-19 prophylactic and therapeutic. At the same time, we speculate that LUAD patients infected with COVID-19 may not be suitable for TMPRSS2 inhibitors treatment.

Taken together, our findings may suggest that:①TMPRSS2 agonists might be an immunomodulator of LUAD patients to anti-PD-1 based immunotherapy combination therapies; ② Gender-dependent AR level difference could be an explanation for the difference in the risk of COVID-19 infection in patients with lung cancer between males and females since the mRNA expression level of AR is significantly positively correlated with TMPRSS2’s; ③Non-TMPRSS2 inhibitor treatment, such as COVID-19 vaccines, should be taken into consideration first in the COVID-19 prophylactic and therapeutic of lung cancer patients, especially LUAD patients.

## Conclusion

In this study, we proposed a possible mechanism that explains why TMPRSS2 expression correlates with immune infiltration leads to different prognosis patterns in different types of lung cancer. These may indicates that TMPRSS2 may be a novel prognostic biomarker for indicating prognostic potential and immune infiltration levels in LUAD and LUSC cohorts and most likely serve as a potential immunomodulator target of immunotherapy combination therapies of nonresponse to anti-PD-1 therapy LUAD patients.

In addition, we identified ginsenosides that can act as TMPRSS2 inhibitors, but due to the different roles of TMPRSS2 expression level in patients with COVID-19 infection and in LUAD patients who do not respond to PD-1 treatment, trade-offs need to be made in medicating TMPRSS2 inhibitors, such as ginsenosides to gain prophylactic and therapeutic benefits against COVID-19 and treatment in LUAD patients.

## Data Availability

The original contributions presented in the study are included in the article/Supplementary Materials, further inquiries can be directed to the corresponding authors.

## Abbreviations

ACE2, angiotensin converting enzyme2; FFPE, paraffin embedded; FP, fluticasone propionate; HR, hazard ratio; LUAD, adenocarcinoma; LUSC, squamous cell carcinoma; NSCLC, non-small cell lung cancer; OS, overall survival; PAMPs, pathogen-associated molecular patterns; PD-1, programmed cell death protein 1; PD-L1, cell surface local programmed cells blockade of death ligand 1; RFS, recurrence-free survival; SCLC, small cell lung cancer; TAMs, tumor-associated macrophages; TILs, tumor-infiltrating lymphocytes; TINs, tumor-infiltrating neutrophils; TLRs, Toll-like receptors; TMPRSS2, transmembrane serine protease 2.
