# Supplementary material for: Ginsenosides, potential TMPRSS2 inhibitors, a trade-off between the therapeutic combination for anti-PD-1 immunotherapy and the treatment of COVID-19 infection of LUAD patients
Source: Front Pharmacol. 2023 Mar 13;14:1085509. doi: 10.3389/fphar.2023.1085509 (PMC10040610; doi:10.3389/fphar.2023.1085509)
Supplement: Supplementary file 1 [file DataSheet2.PDF]

SI-Table 2. Relation between TMPRSS2 expression and the clinicopathological parameters in LUAD and LUSC

LUAD		LUSC	
**Individual cancer stages**		**Individual cancer stages**	
**Comparison**	**Statistical significance**	**Comparison**	**Statistical significance**
Normal-vs-Stage1	**7.72E-07**	Normal-vs-Stage1	**1.62E-12**
Normal-vs-Stage2	**6.40E-04**	Normal-vs-Stage2	**<1E-12**
Normal-vs-Stage3	**2.80E-12**	Normal-vs-Stage3	**1.62E-12**
Normal-vs-Stage4	**1.64E-04**	Normal-vs-Stage4	**3.44E-12**
Stage1-vs-Stage2	4.59E-01	Stage1-vs-Stage2	8.34E-02
Stage1-vs-Stage3	**2.40E-04**	Stage1-vs-Stage3	7.56E-01
Stage1-vs-Stage4	9.28E-02	Stage1-vs-Stage4	4.97E-01
Stage2-vs-Stage3	1.46E-01	Stage2-vs-Stage3	4.58E-01
Stage2-vs-Stage4	3.83E-01	Stage2-vs-Stage4	6.97E-01
Stage3-vs-Stage4	0.7744	Stage3-vs-Stage4	0.38966
**Patient's Race**		**Patient's Race**	
**Comparison**	**Statistical significance**	**Comparison**	**Statistical significance**
Normal-vs-Caucasian	**6.20E-08**	Normal-vs-Caucasian	**1.62E-12**
Normal-vs-AfricanAmerican	**1.73E-06**	Normal-vs-AfricanAmerican	**1.62E-12**
Normal-vs-Asian	4.70E-01	Normal-vs-Asian	**3.68E-05**
Caucasian-vs-AfricanAmerican	1.84E-01	Caucasian-vs-AfricanAmerican	2.98E-01
Caucasian-vs-Asian	6.27E-02	Caucasian-vs-Asian	3.83E-01
AfricanAmerican-vs-Asian	**5.12E-03**	AfricanAmerican-vs-Asian	2.93E-01
**Patient's Gender**		**Patient's Gender**	
**Comparison**	**Statistical significance**	**Comparison**	**Statistical significance**
Normal-vs-Male	**1.81E-12**	Normal-vs-Male	**<1E-12**
Normal-vs-Female	**1.76E-04**	Normal-vs-Female	**<1E-12**
Male-vs-Female	**1.06E-03**	Male-vs-Female	8.69E-02
**Patient's age**		**Patient's age**	
**Comparison**	**Statistical significance**	**Comparison**	**Statistical significance**
Normal-vs-Age(21-40Yrs)	**2.41E-05**	Normal-vs-Age(21-40Yrs)	**1.42E-02**
Normal-vs-Age(41-60Yrs)	**5.11E-10**	Normal-vs-Age(41-60Yrs)	**1.62E-12**
Normal-vs-Age(61-80Yrs)	**2.43E-06**	Normal-vs-Age(61-80Yrs)	**<1E-12**
Normal-vs-Age(81-100Yrs)	**3.55E-05**	Normal-vs-Age(81-100Yrs)	**1.62E-12**
Age(21-40Yrs)-vs-Age(41-60Yrs)	0.180216	Age(21-40Yrs)-vs-Age(41-60Yrs)	0.88342
Age(21-40Yrs)-vs-Age(61-80Yrs)	0.21952	Age(21-40Yrs)-vs-Age(61-80Yrs)	0.70162
Age(21-40Yrs)-vs-Age(81-100Yrs)	0.103417	Age(21-40Yrs)-vs-Age(81-100Yrs)	0.69638
Age(41-60Yrs)-vs-Age(61-80Yrs)	0.07908	Age(41-60Yrs)-vs-Age(61-80Yrs)	0.51624
Age(41-60Yrs)-vs-Age(81-100Yrs)	0.87412	Age(41-60Yrs)-vs-Age(81-100Yrs)	0.067417
Age(61-80Yrs)-vs-Age(81-100Yrs)	0.34962	Age(61-80Yrs)-vs-Age(81-100Yrs)	0.058079
**Patient's smoking habit**		**Patient's smoking habit**	
**Comparison**	**Statistical significance**	**Comparison**	**Statistical significance**
Normal-vs-Non smoker	**1.26E-02**	Normal-vs-Non smoker	**2.88E-10**
Normal-vs-Smoker	**4.44E-05**	Normal-vs-Smoker	**1.62E-12**
Normal-vs-Reformed smoker1	**1.75E-02**	Normal-vs-Reformed smoker1	**1.62E-12**
Normal-vs-Reformed smoker2	**2.27E-13**	Normal-vs-Reformed smoker2	**<1E-12**
Non smoker-vs-Smoker	**3.94E-02**	Non smoker-vs-Smoker	6.93E-01
Non smoker-vs-Reformed smoker1	6.66E-01	Non smoker-vs-Reformed smoker1	6.14E-01
Non smoker-vs-Reformed smoker2	**3.20E-04**	Non smoker-vs-Reformed smoker2	7.37E-01
Smoker-vs-Reformed smoker1	**1.17E-02**	Smoker-vs-Reformed smoker1	6.59E-01
Smoker-vs-Reformed smoker2	8.92E-01	Smoker-vs-Reformed smoker2	9.77E-01
Reformed smoker1-vs-Reformed smoker2	**3.30E-06**	Reformed smoker1-vs-Reformed smoker2	5.50E-01

Bold numbers indicate statistical significance, $*P \leqslant 0.05$, $\#P \leqslant 0.001$, $\triangle P \leqslant 0.0001$
