# Supplementary figures and images for: Ginsenosides, potential TMPRSS2 inhibitors, a trade-off between the therapeutic combination for anti-PD-1 immunotherapy and the treatment of COVID-19 infection of LUAD patients
Source: Front Pharmacol. 2023 Mar 13;14:1085509. doi: 10.3389/fphar.2023.1085509 (PMC10040610; doi:10.3389/fphar.2023.1085509)

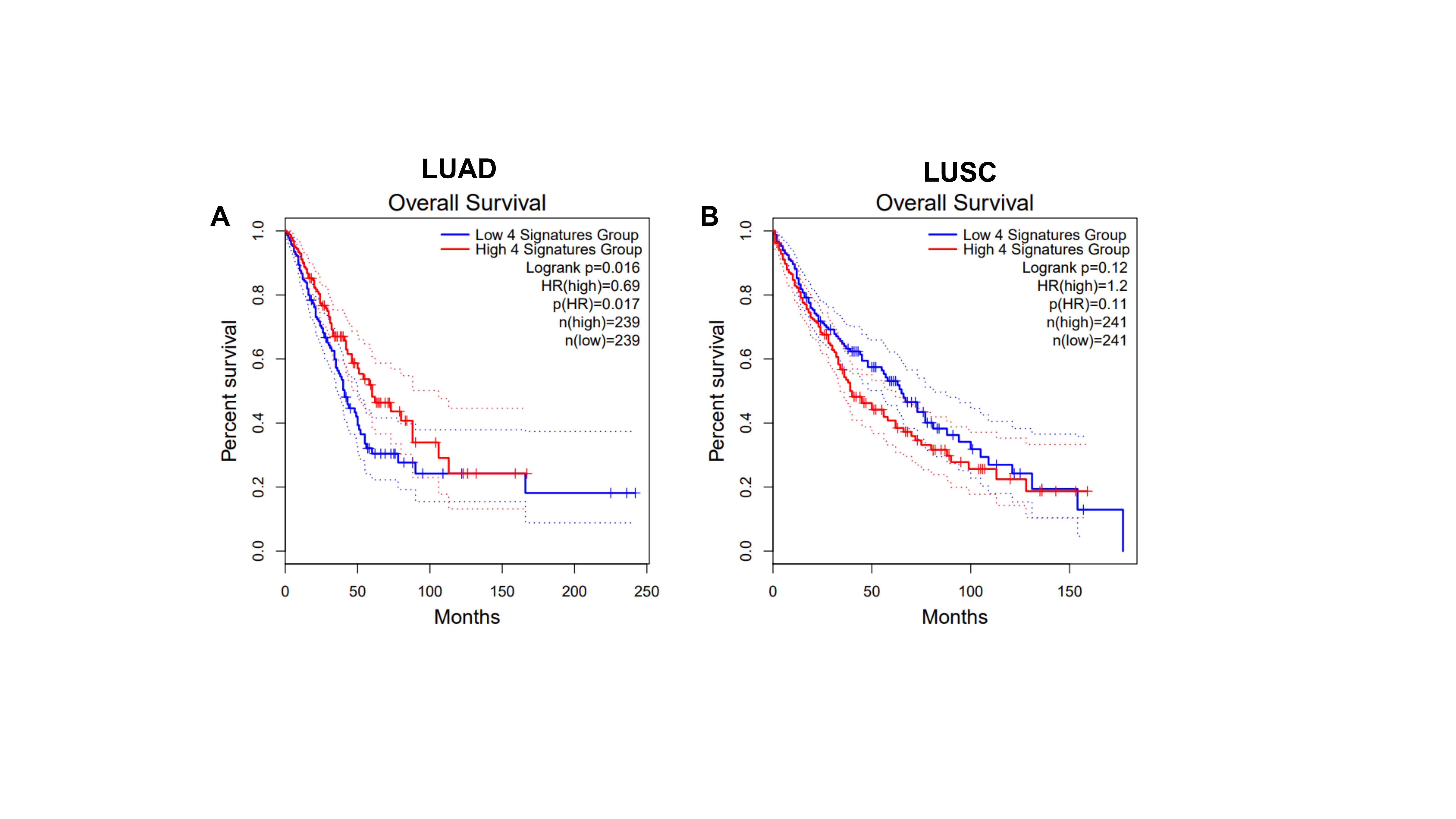

Supplement: Supplementary file 2 [file Image3.JPEG]

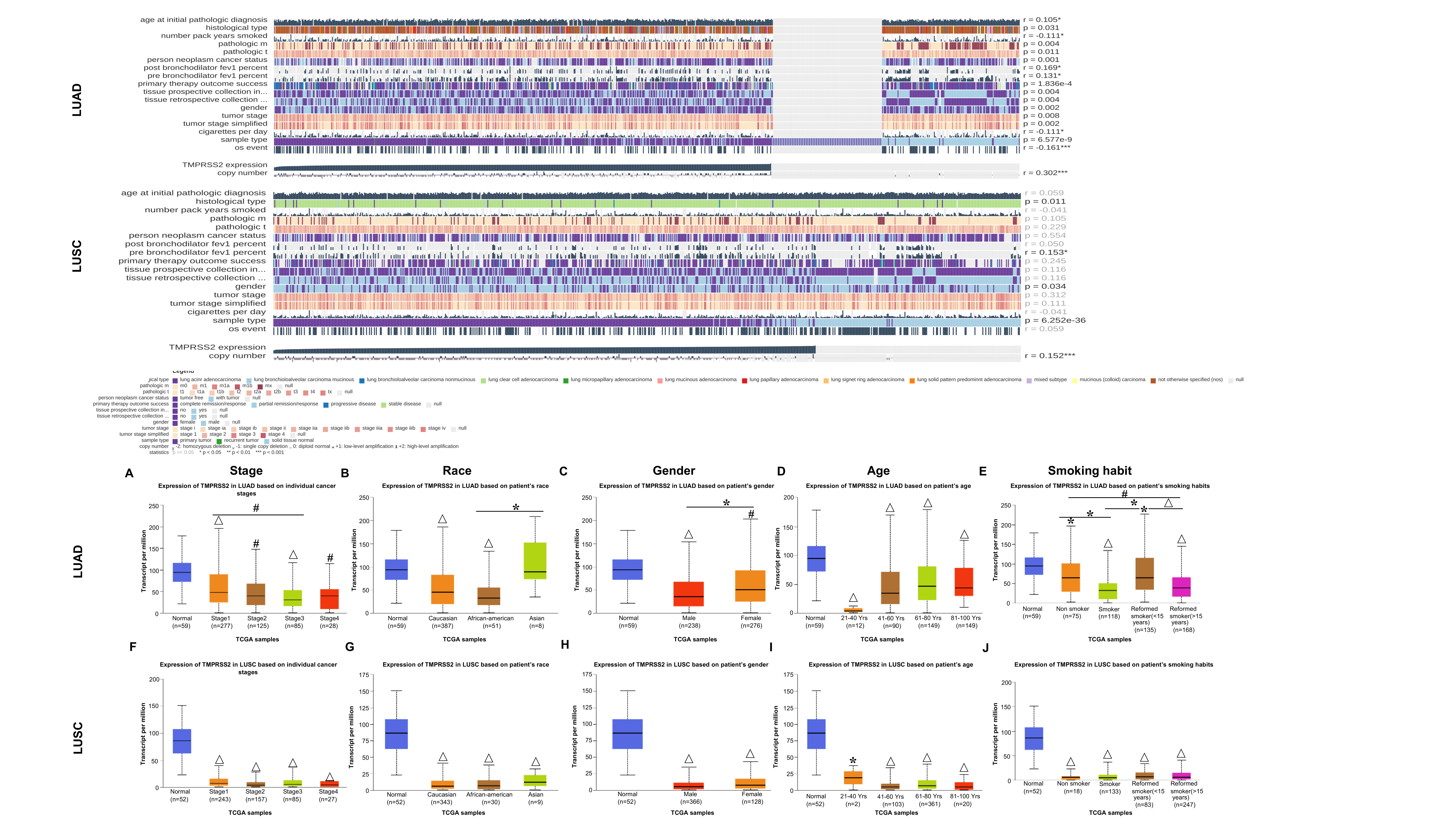

Supplement: Supplementary file 5 [file Image1.JPEG]

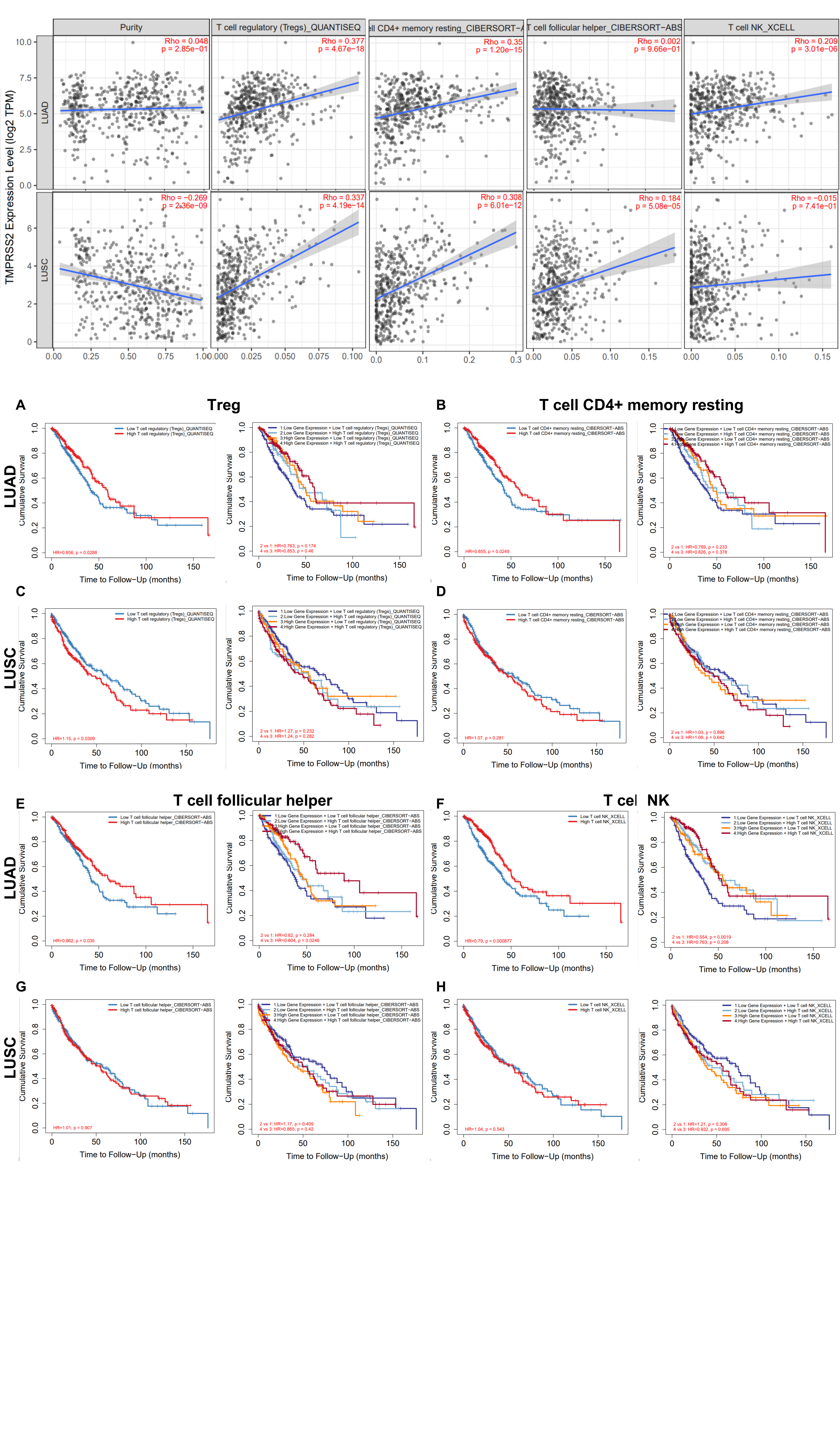

Supplement: Supplementary file 6 [file Image4.JPEG]

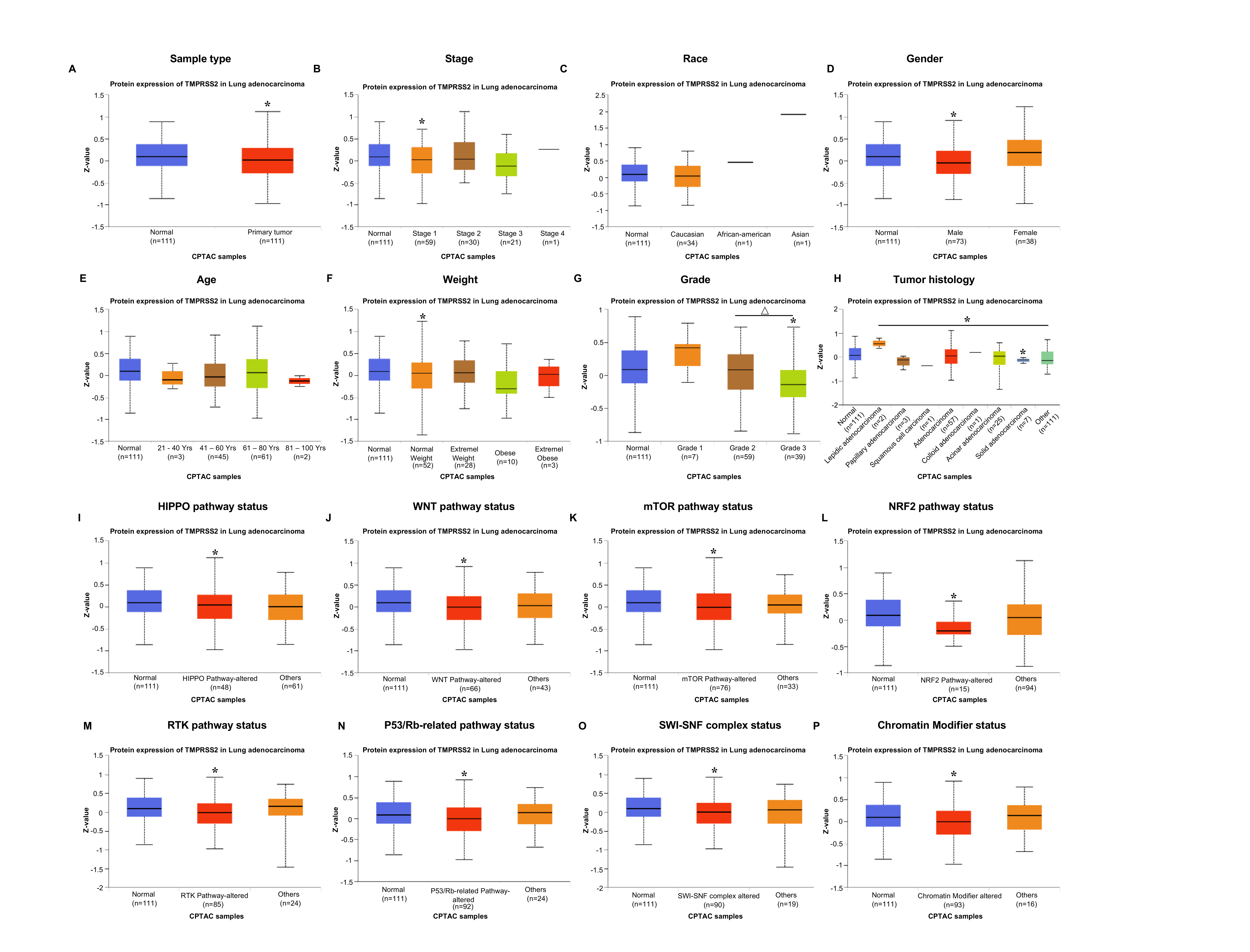

Supplement: Supplementary file 7 [file Image2.JPEG]

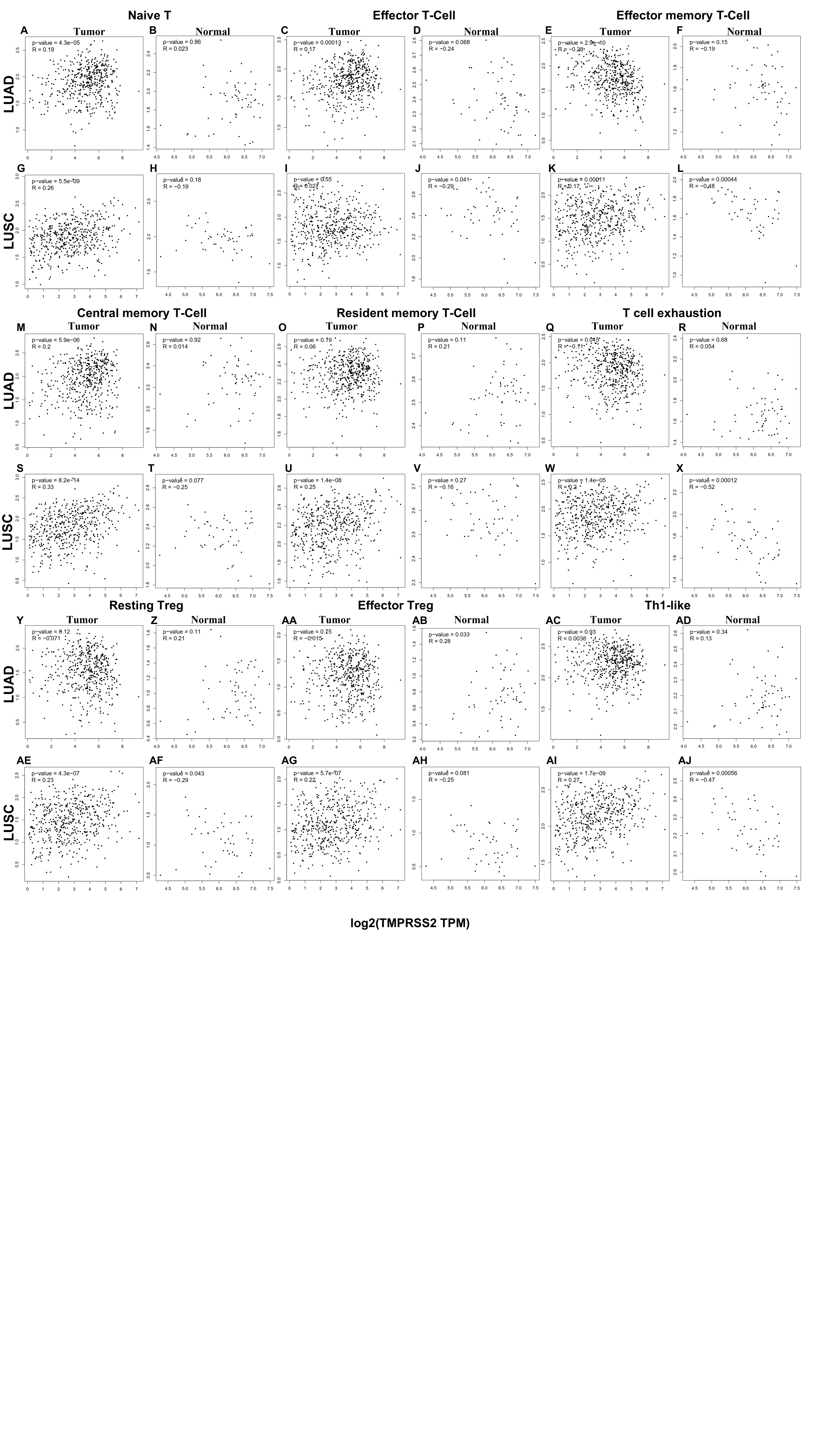

Supplement: Supplementary file 8 [file Image5.JPEG]
