# Supplementary material for: Ginsenosides, potential TMPRSS2 inhibitors, a trade-off between the therapeutic combination for anti-PD-1 immunotherapy and the treatment of COVID-19 infection of LUAD patients
Source: Front Pharmacol. 2023 Mar 13;14:1085509. doi: 10.3389/fphar.2023.1085509 (PMC10040610; doi:10.3389/fphar.2023.1085509)
Supplement: Supplementary file 3 [file DataSheet4.PDF]

SI- Table 4. Information on methylation site changes of TMPRSS2 in LUAD and LUSC in MEXPRESS database

CHR	CpG location	LUAD	LUSC	Probe ID	Probe start	Probe end	Design type	CpG count	Sequence around CpG
21	41465036	**r = 0.205*****	r = 0.017	cg18156003	41464987	41465036	II	1	ATTGGAAGGGACCACAGAGAGTCCAAAACAAAACACATCTTTCTCTTCTTCGCCGCCACCATGGGCACTTTGCTTCAGCACATTCATTTATAGAACGTTAA
	41467699	r = -0.082	r = 0.062	cg19974120	41467699	41467748	II	1	TTATCGACAAATGAGGGTAACTATCCTGTCCTCCTTCTGACTGTGTTCTCCGATTCCTCGAGCCAAAGCCAGACATCTGTTAGGCGTGGTTCTGCTGCTGG
	41488290	**r = 0.098***	**r = 0.243*****	cg19533416	41488241	41488290	II	1	GAGCCCCAGGGCTCAGCCTGGAGCAGGGATTCAATGACATCATGTGTCAGCGTACTGGACGCACGCCAGGCCCAGTCACCCAAAGGCCCCTGGACCCGG
	41504034	**r = -0.453*****	r = -0.072	cg13489049	41503985	41504034	II	1	CCTTGGAAAGCAATGGCCTTAAAGGGAATTTGACACTGCATTGTGCACCTCGTACAAACAAGCAATAATAAAAAGCCTCAGCTTAGTTCCATCTGCCAAGT
	41505711	**r = -0.491*****	**r = -0.298*****	cg01157146	41505662	41505711	II	1	GGGGAGAACCAGAGCCTGGCCCAGGAACAAGCCCTTTTCAATCAGGATTCCGGGCGCCTTGTTTCCAGCTTCCTTCCCTCCTTTCCTCCACTTGCTGGTAA
	41507148	**r = -0.354*****	**r = -0.264*****	cg16084872	41507148	41507197	II	2	CTCGAGCACGGGTCCAGCTGAAAAACTGCCCCATGTCCAGAAAGTTTGTGCGCCGAGAGCCCTGAGCGCTTGTGGCAGAGGAGGGCCTTCGCCCGCTCCG
	41507205	**r = -0.297*****	**r = -0.241*****	cg02613803	41507205	41507254	II	1	CTCGGCACAGTGGCACCCTGAGTGGTCAAGTGCTCCAGTCTGGCAGTGGGCGCTCTGCTCGAGCACGGGTCCAGCTGAAAAACTGCCCCATGTCCAGAAA
	41507286	r = -0.030	r = -0.006	cg16051245	41507238	41507287	I	4	CAGGGTGCCACTGTGCCGAGCCGGGCAGGACAGGATGAGGTGGACCGAAGCGCCCAGGTGCCCCGCCTTCCCCCCAACCCGGCACGGAGCGCAAGCGG
	41507732	r = 0.080	r = -0.055	cg00739644	41507732	41507781	II	1	CCAGTTGGTGGAAGAGAGTCCCCCGGGGTCCCCAAAGCTGGCTCCTAGTCCGCCTGCCCTCCACGGCCCCCGCCTGGGAGCACCGGTGCGCCTTTTCTCTT
	41508072	r = -0.014	r = 0.075	cg26337277	41508071	41508120	I	8	GCGGAGGGCGAGGGGCGGGGAGCGCCGCCTGGAGCGCGGCAGGTGAGCGGCGCCGGTACCAGGGTCCCGGCTCGGGGTCCGGGCTGGGGAGGGGAA
	41508181	r = -0.058	**r = 0.143****	cg00689211	41508181	41508230	II	2	GCTTGGGGGTGTCGCCCTGGACCCTGGGACACCGCCTCCTGAGATTAAAGCGAGAGCCAGGGCGGGCCGGGCCGAGTAGGCGCGAGCTAAGCAGGAGG
	41508522	**r = -0.372*****	**r = -0.249*****	cg16371860	41508521	41508570	I	3	CCTTGCCCAGAGGCTACAGTGGGTTCCCCGGAGGCCAAGACGGGGCCGGCCGCCTACAGGAGCTCGTGAGGTAGCAGCTCCGGGGGCTCACCCAGGACT
	41508627	**r = -0.477*****	**r = -0.300*****	cg24901042	41508578	41508627	II	3	AGGGTAGGCCTTGGGGACTGCTGGCCCAAGCACGAGTGGCGGGGACACAGCGCCGGACGCCCAAGACCCTCCCCAGTCCGCCTAGGCTGGCATTTCTTG
	41508630	**r = -0.464*****	**r = -0.318*****	cg26309194	41508582	41508631	I	4	GTAGGCCTTGGGGACTGCTGGCCCAAGCACGAGTGGCGGGGACACAGCGCCGGACGCCCAAGACCCTCCCCAGTCCGCCTAGGCTGGCATTTCTTGGCCC
	41508656	**r = -0.449*****	**r = -0.314*****	cg14982276	41508655	41508704	I	2	GGAGCACTTTCCACCGGACGCTGGTGGGGGCCAAGAAATGCCAGCCTAGGCGGACTGGGGAGGGTCTTGGGCGTCCGGCGCTGTGTCCCCGCCACTCGT
	41508788	**r = -0.470*****	**r = -0.305*****	cg12384236	41508788	41508837	II	1	CCACAATTGCAAACATTAGAAAGAACCTCTCAAGTGCCCGGGAACAGCCACGTCTTCCTGGCTGAGGTGTGTCCCACCACTTCCTCACTCCCGCCCTGGCC

Bold numbers indicate statistical significance.
