# Supplementary material for: Ginsenosides, potential TMPRSS2 inhibitors, a trade-off between the therapeutic combination for anti-PD-1 immunotherapy and the treatment of COVID-19 infection of LUAD patients
Source: Front Pharmacol. 2023 Mar 13;14:1085509. doi: 10.3389/fphar.2023.1085509 (PMC10040610; doi:10.3389/fphar.2023.1085509)
Supplement: Supplementary file 4 [file DataSheet6.PDF]

SI-Table 6. Top and bottom 100 of IPS of LUAD and LUSC patients who were likely to responde to anti-PD-1 therapy

Patient	Disease	Gender	Age	Study	IPS
1	LUAD	male	68	TCGA	10
2	LUAD	male	75	TCGA	10
3	LUAD	male	60	TCGA	10
4	LUAD	male	62	TCGA	10
5	LUAD	female	79	TCGA	9
6	LUAD	female	73	TCGA	9
7	LUAD	female	70	TCGA	9
8	LUAD	female	75	TCGA	9
9	LUAD	female	68	TCGA	9
10	LUAD	female	65	TCGA	9
11	LUAD	female	77	TCGA	9
12	LUAD	female	42	TCGA	9
13	LUAD	female	63	TCGA	9
14	LUAD	female	55	TCGA	9
15	LUAD	female	73	TCGA	9
16	LUAD	female	60	TCGA	9
17	LUAD	male	70	TCGA	9
18	LUAD	female	84	TCGA	9
19	LUAD	female	73	TCGA	9
20	LUAD	male	63	TCGA	9
21	LUAD	male	68	TCGA	9
22	LUAD	male	NA	TCGA	9
23	LUAD	female	54	TCGA	9
24	LUAD	female	74	TCGA	9
25	LUAD	female	60	TCGA	9
26	LUAD	female	72	TCGA	9
27	LUAD	male	84	TCGA	9
28	LUAD	female	81	TCGA	9
29	LUAD	female	77	TCGA	9
30	LUAD	male	64	TCGA	9
31	LUAD	female	79	TCGA	9
32	LUAD	male	56	TCGA	9
33	LUAD	female	68	TCGA	9
34	LUAD	male	59	TCGA	9
35	LUAD	female	66	TCGA	9
36	LUAD	female	59	TCGA	9
37	LUAD	male	71	TCGA	9
38	LUAD	female	55	TCGA	9
39	LUAD	female	73	TCGA	9
40	LUAD	female	72	TCGA	9
41	LUAD	male	NA	TCGA	9
42	LUAD	male	67	TCGA	9
43	LUAD	female	72	TCGA	9
44	LUAD	female	62	TCGA	9
45	LUAD	male	81	TCGA	9
46	LUAD	female	50	TCGA	9
47	LUAD	male	58	TCGA	9
48	LUAD	female	79	TCGA	9
49	LUAD	male	81	TCGA	8
50	LUAD	male	55	TCGA	8
51	LUAD	male	63	TCGA	8
52	LUAD	male	70	TCGA	8
53	LUAD	male	75	TCGA	8
54	LUAD	female	65	TCGA	8
55	LUAD	male	76	TCGA	8

High-scoring group of LUAD patient cohorts

56	LUAD	female	73	TCGA	8
57	LUAD	male	71	TCGA	8
58	LUAD	female	74	TCGA	8
59	LUAD	male	74	TCGA	8
60	LUAD	female	75	TCGA	8
61	LUAD	female	86	TCGA	8
62	LUAD	female	NA	TCGA	8
63	LUAD	male	75	TCGA	8
64	LUAD	male	80	TCGA	8
65	LUAD	male	NA	TCGA	8
66	LUAD	female	68	TCGA	8
67	LUAD	male	74	TCGA	8
68	LUAD	female	66	TCGA	8
69	LUAD	female	67	TCGA	8
70	LUAD	female	65	TCGA	8
71	LUAD	female	61	TCGA	8
72	LUAD	female	59	TCGA	8
73	LUAD	male	42	TCGA	8
74	LUAD	male	53	TCGA	8
75	LUAD	male	75	TCGA	8
76	LUAD	male	74	TCGA	8
77	LUAD	male	72	TCGA	8
78	LUAD	male	64	TCGA	8
79	LUAD	female	56	TCGA	8
80	LUAD	female	61	TCGA	8
81	LUAD	male	74	TCGA	8
82	LUAD	male	77	TCGA	8
83	LUAD	female	52	TCGA	8
84	LUAD	male	64	TCGA	8
85	LUAD	male	65	TCGA	8
86	LUAD	male	NA	TCGA	8
87	LUAD	male	67	TCGA	8
88	LUAD	male	43	TCGA	8
89	LUAD	female	65	TCGA	8
90	LUAD	female	72	TCGA	8
91	LUAD	female	70	TCGA	8
92	LUAD	male	63	TCGA	8
93	LUAD	female	73	TCGA	8
94	LUAD	female	72	TCGA	8
95	LUAD	male	60	TCGA	8
96	LUAD	male	72	TCGA	8
97	LUAD	male	60	TCGA	8
98	LUAD	female	NA	TCGA	8
99	LUAD	female	82	TCGA	8
100	LUAD	male	72	TCGA	8
101	LUAD	female	67	TCGA	6
102	LUAD	female	65	TCGA	6
103	LUAD	female	72	TCGA	6
104	LUAD	male	41	TCGA	6
105	LUAD	female	62	TCGA	6
106	LUAD	female	63	TCGA	6
107	LUAD	male	67	TCGA	6
108	LUAD	female	77	TCGA	6
109	LUAD	female	69	TCGA	6
110	LUAD	female	57	TCGA	6
111	LUAD	male	69	TCGA	6
112	LUAD	male	63	TCGA	6
113	LUAD	female	76	TCGA	6

114	LUAD	female	69	TCGA	6	
115	LUAD	female	87	TCGA	6	
116	LUAD	female	NA	TCGA	6	
117	LUAD	male	58	TCGA	6	
118	LUAD	male	78	TCGA	6	
119	LUAD	male	68	TCGA	6	
120	LUAD	female	78	TCGA	6	
121	LUAD	female	72	TCGA	6	
122	LUAD	female	53	TCGA	6	
123	LUAD	female	66	TCGA	6	
124	LUAD	male	79	TCGA	6	
125	LUAD	female	62	TCGA	6	
126	LUAD	male	66	TCGA	6	
127	LUAD	female	75	TCGA	6	
128	LUAD	female	74	TCGA	6	
129	LUAD	male	NA	TCGA	6	
130	LUAD	female	42	TCGA	6	
131	LUAD	female	56	TCGA	6	
132	LUAD	male	76	TCGA	6	
133	LUAD	male	68	TCGA	6	
134	LUAD	female	69	TCGA	6	
135	LUAD	male	60	TCGA	6	
136	LUAD	female	53	TCGA	6	
137	LUAD	male	77	TCGA	6	
138	LUAD	male	60	TCGA	6	
139	LUAD	male	60	TCGA	6	
140	LUAD	male	52	TCGA	6	
141	LUAD	female	52	TCGA	6	
142	LUAD	male	61	TCGA	6	
143	LUAD	female	58	TCGA	6	
144	LUAD	male	48	TCGA	6	
145	LUAD	male	75	TCGA	6	
146	LUAD	male	70	TCGA	6	
147	LUAD	female	70	TCGA	6	
148	LUAD	female	60	TCGA	6	
149	LUAD	female	71	TCGA	6	
150	LUAD	male	74	TCGA	6	Low-scoring group of LUAD patient cohorts
151	LUAD	female	61	TCGA	6	
152	LUAD	male	61	TCGA	6	
153	LUAD	male	62	TCGA	6	
154	LUAD	male	53	TCGA	6	
155	LUAD	male	58	TCGA	6	
156	LUAD	male	56	TCGA	6	
157	LUAD	male	NA	TCGA	6	
158	LUAD	male	73	TCGA	6	
159	LUAD	female	69	TCGA	6	
160	LUAD	male	56	TCGA	6	
161	LUAD	male	66	TCGA	6	
162	LUAD	female	51	TCGA	6	
163	LUAD	male	73	TCGA	6	
164	LUAD	male	70	TCGA	6	
165	LUAD	male	69	TCGA	6	
166	LUAD	male	66	TCGA	6	
167	LUAD	female	54	TCGA	6	
168	LUAD	female	69	TCGA	6	
169	LUAD	male	62	TCGA	6	
170	LUAD	male	57	TCGA	6	
171	LUAD	female	81	TCGA	6	

172	LUAD	female	NA	TCGA	6
173	LUAD	male	72	TCGA	6
174	LUAD	female	71	TCGA	6
175	LUAD	male	45	TCGA	5
176	LUAD	male	62	TCGA	5
177	LUAD	male	61	TCGA	5
178	LUAD	female	51	TCGA	5
179	LUAD	male	46	TCGA	5
180	LUAD	male	67	TCGA	5
181	LUAD	female	53	TCGA	5
182	LUAD	female	63	TCGA	5
183	LUAD	female	70	TCGA	5
184	LUAD	female	78	TCGA	5
185	LUAD	male	65	TCGA	5
186	LUAD	female	50	TCGA	5
187	LUAD	male	59	TCGA	5
188	LUAD	female	65	TCGA	5
189	LUAD	male	84	TCGA	5
190	LUAD	male	60	TCGA	5
191	LUAD	male	57	TCGA	5
192	LUAD	male	68	TCGA	5
193	LUAD	female	60	TCGA	5
194	LUAD	female	49	TCGA	5
195	LUAD	female	58	TCGA	5
196	LUAD	male	78	TCGA	5
197	LUAD	female	59	TCGA	5
198	LUAD	female	50	TCGA	5
199	LUAD	male	70	TCGA	5
200	LUAD	male	65	TCGA	5
201	LUSC	female	65	TCGA	9
202	LUSC	female	66	TCGA	9
203	LUSC	male	63	TCGA	9
204	LUSC	male	59	TCGA	9
205	LUSC	male	74	TCGA	9
206	LUSC	male	73	TCGA	9
207	LUSC	male	44	TCGA	9
208	LUSC	male	81	TCGA	9
209	LUSC	female	78	TCGA	9
210	LUSC	male	59	TCGA	9
211	LUSC	male	61	TCGA	9
212	LUSC	male	83	TCGA	9
213	LUSC	male	73	TCGA	9
214	LUSC	male	60	TCGA	9
215	LUSC	male	68	TCGA	9
216	LUSC	male	64	TCGA	9
217	LUSC	male	71	TCGA	9
218	LUSC	female	72	TCGA	9
219	LUSC	female	67	TCGA	9
220	LUSC	male	76	TCGA	9
221	LUSC	male	53	TCGA	9
222	LUSC	male	75	TCGA	9
223	LUSC	male	76	TCGA	9
224	LUSC	male	65	TCGA	9
225	LUSC	male	64	TCGA	8
226	LUSC	male	73	TCGA	8
227	LUSC	female	74	TCGA	8
228	LUSC	female	60	TCGA	8
229	LUSC	male	63	TCGA	8

230	LUSC	male	79	TCGA	8	
231	LUSC	female	65	TCGA	8	
232	LUSC	female	62	TCGA	8	
233	LUSC	female	72	TCGA	8	
234	LUSC	male	41	TCGA	8	
235	LUSC	male	56	TCGA	8	
236	LUSC	male	73	TCGA	8	
237	LUSC	male	75	TCGA	8	
238	LUSC	male	69	TCGA	8	
239	LUSC	male	72	TCGA	8	
240	LUSC	female	62	TCGA	8	
241	LUSC	male	64	TCGA	8	
242	LUSC	male	74	TCGA	8	
243	LUSC	female	74	TCGA	8	
244	LUSC	male	78	TCGA	8	
245	LUSC	male	71	TCGA	8	
246	LUSC	male	70	TCGA	8	
247	LUSC	female	60	TCGA	8	
248	LUSC	female	71	TCGA	8	
249	LUSC	male	NA	TCGA	8	
250	LUSC	male	65	TCGA	8	High-scoring group of LUSC patient cohorts
251	LUSC	female	61	TCGA	8	
252	LUSC	male	62	TCGA	8	
253	LUSC	male	61	TCGA	8	
254	LUSC	female	81	TCGA	8	
255	LUSC	female	58	TCGA	8	
256	LUSC	male	62	TCGA	8	
257	LUSC	male	70	TCGA	8	
258	LUSC	male	67	TCGA	8	
259	LUSC	female	78	TCGA	8	
260	LUSC	male	64	TCGA	8	
261	LUSC	male	66	TCGA	8	
262	LUSC	male	48	TCGA	8	
263	LUSC	male	53	TCGA	8	
264	LUSC	male	77	TCGA	8	
265	LUSC	female	69	TCGA	8	
266	LUSC	male	56	TCGA	8	
267	LUSC	male	66	TCGA	8	
268	LUSC	male	56	TCGA	8	
269	LUSC	male	66	TCGA	8	
270	LUSC	female	79	TCGA	8	
271	LUSC	male	61	TCGA	8	
272	LUSC	male	60	TCGA	8	
273	LUSC	male	78	TCGA	8	
274	LUSC	male	58	TCGA	8	
275	LUSC	male	47	TCGA	8	
276	LUSC	male	68	TCGA	8	
277	LUSC	male	72	TCGA	8	
278	LUSC	male	64	TCGA	8	
279	LUSC	male	63	TCGA	8	
280	LUSC	male	66	TCGA	8	
281	LUSC	female	66	TCGA	8	
282	LUSC	male	40	TCGA	8	
283	LUSC	male	65	TCGA	8	
284	LUSC	male	69	TCGA	8	
285	LUSC	male	64	TCGA	8	
286	LUSC	male	70	TCGA	8	
287	LUSC	male	71	TCGA	8	

288	LUSC	male	51	TCGA	8
289	LUSC	female	76	TCGA	8
290	LUSC	female	60	TCGA	8
291	LUSC	female	61	TCGA	8
292	LUSC	male	71	TCGA	8
293	LUSC	male	70	TCGA	8
294	LUSC	female	70	TCGA	8
295	LUSC	female	65	TCGA	8
296	LUSC	female	77	TCGA	8
297	LUSC	male	79	TCGA	8
298	LUSC	male	71	TCGA	8
299	LUSC	male	63	TCGA	8
300	LUSC	male	59	TCGA	8
301	LUSC	male	70	TCGA	6
302	LUSC	male	73	TCGA	6
303	LUSC	male	68	TCGA	6
304	LUSC	male	69	TCGA	6
305	LUSC	male	67	TCGA	6
306	LUSC	male	67	TCGA	6
307	LUSC	female	83	TCGA	6
308	LUSC	male	57	TCGA	6
309	LUSC	male	71	TCGA	6
310	LUSC	female	52	TCGA	6
311	LUSC	male	72	TCGA	6
312	LUSC	male	62	TCGA	6
313	LUSC	male	NA	TCGA	6
314	LUSC	female	73	TCGA	6
315	LUSC	female	57	TCGA	6
316	LUSC	male	69	TCGA	6
317	LUSC	female	76	TCGA	6
318	LUSC	female	59	TCGA	6
319	LUSC	male	58	TCGA	6
320	LUSC	male	64	TCGA	6
321	LUSC	female	47	TCGA	6
322	LUSC	female	55	TCGA	6
323	LUSC	male	66	TCGA	6
324	LUSC	male	76	TCGA	6
325	LUSC	male	68	TCGA	6
326	LUSC	female	57	TCGA	6
327	LUSC	male	67	TCGA	6
328	LUSC	female	72	TCGA	6
329	LUSC	male	54	TCGA	6
330	LUSC	male	60	TCGA	6
331	LUSC	male	46	TCGA	6
332	LUSC	male	67	TCGA	6
333	LUSC	male	69	TCGA	6
334	LUSC	female	67	TCGA	6
335	LUSC	female	75	TCGA	6
336	LUSC	male	72	TCGA	6
337	LUSC	male	63	TCGA	6
338	LUSC	male	75	TCGA	6
339	LUSC	male	74	TCGA	6
340	LUSC	male	80	TCGA	6
341	LUSC	male	70	TCGA	6
342	LUSC	male	68	TCGA	6
343	LUSC	male	65	TCGA	6
344	LUSC	male	47	TCGA	6
345	LUSC	male	58	TCGA	6

346	LUSC	male	59	TCGA	6	
347	LUSC	female	77	TCGA	5	
348	LUSC	male	64	TCGA	5	
349	LUSC	male	65	TCGA	5	Low-scoring group of LUSC patient cohorts
350	LUSC	male	55	TCGA	5	
351	LUSC	male	52	TCGA	5	
352	LUSC	female	70	TCGA	5	
353	LUSC	male	68	TCGA	5	
354	LUSC	male	68	TCGA	5	
355	LUSC	male	63	TCGA	5	
356	LUSC	male	55	TCGA	5	
357	LUSC	male	61	TCGA	5	
358	LUSC	female	69	TCGA	5	
359	LUSC	female	62	TCGA	5	
360	LUSC	male	74	TCGA	5	
361	LUSC	male	77	TCGA	5	
362	LUSC	female	78	TCGA	5	
363	LUSC	male	81	TCGA	5	
364	LUSC	male	60	TCGA	5	
365	LUSC	male	71	TCGA	5	
366	LUSC	male	67	TCGA	5	
367	LUSC	male	66	TCGA	5	
368	LUSC	female	52	TCGA	5	
369	LUSC	female	70	TCGA	5	
370	LUSC	female	60	TCGA	5	
371	LUSC	male	68	TCGA	5	
372	LUSC	female	76	TCGA	5	
373	LUSC	male	NA	TCGA	5	
374	LUSC	male	69	TCGA	5	
375	LUSC	male	79	TCGA	5	
376	LUSC	male	70	TCGA	5	
377	LUSC	male	68	TCGA	5	
378	LUSC	male	57	TCGA	5	
379	LUSC	male	63	TCGA	5	
380	LUSC	male	79	TCGA	5	
381	LUSC	female	71	TCGA	5	
382	LUSC	male	66	TCGA	5	
383	LUSC	male	70	TCGA	5	
384	LUSC	male	74	TCGA	5	
385	LUSC	male	66	TCGA	5	
386	LUSC	male	81	TCGA	5	
387	LUSC	male	83	TCGA	5	
388	LUSC	male	73	TCGA	5	
389	LUSC	male	66	TCGA	5	
390	LUSC	male	71	TCGA	5	
391	LUSC	male	73	TCGA	5	
392	LUSC	female	45	TCGA	5	
393	LUSC	male	66	TCGA	4	
394	LUSC	male	69	TCGA	4	
395	LUSC	male	73	TCGA	4	
396	LUSC	male	70	TCGA	4	
397	LUSC	male	53	TCGA	4	
398	LUSC	male	61	TCGA	4	
399	LUSC	female	68	TCGA	4	
400	LUSC	male	63	TCGA	3	
