# Supplementary material for: Ginsenosides, potential TMPRSS2 inhibitors, a trade-off between the therapeutic combination for anti-PD-1 immunotherapy and the treatment of COVID-19 infection of LUAD patients
Source: Front Pharmacol. 2023 Mar 13;14:1085509. doi: 10.3389/fphar.2023.1085509 (PMC10040610; doi:10.3389/fphar.2023.1085509)
Supplement: Supplementary file 9 [file DataSheet3.PDF]

SI- Table 3. Relation between Protein level of TMPRSS2 and the clinicopathological parameters of LUAD

Clinical parameters	P-value
**Sample Types**	
Normal-vs-Primary	**1.37E-02**
**Stage**	
Normal-vs-Stage1	**2.10E-02**
Normal-vs-Stage2	6.07E-01
Normal-vs-Stage3	1.55E-01
Normal-vs-Stage4	NA
Stage1-vs-Stage2	1.32E-01
Stage1-vs-Stage3	3.16E-01
Stage1-vs-Stage4	NA
Stage2-vs-Stage3	5.28E-01
Stage2-vs-Stage4	NA
Stage3-vs-Stage4	NA
**Race**	
Normal-vs-Caucasian	1.05E-01
Normal-vs-AfricanAmerican	NA
Normal-vs-Asian	NA
Caucasian-vs-AfricanAmerican	NA
Caucasian-vs-Asian	NA
AfricanAmerican-vs-Asian	NA
**Gender**	
Normal-vs-Male	**8.48E-03**
Normal-vs-Female	2.36E-01
Male-vs-Female	8.94E-01
**Age**	
Normal-vs-Age(21-40Yrs)	4.78E-01
Normal-vs-Age(41-60Yrs)	1.03E-01
Normal-vs-Age(61-80Yrs)	5.06E-02
Normal-vs-Age(81-100Yrs)	2.86E-01
Age(21-40Yrs)-vs-Age(41-60Yrs)	8.34E-01
Age(21-40Yrs)-vs-Age(61-80Yrs)	4.73E-01
Age(21-40Yrs)-vs-Age(81-100Yrs)	7.07E-01
Age(41-60Yrs)-vs-Age(61-80Yrs)	4.94E-01
Age(41-60Yrs)-vs-Age(81-100Yrs)	8.15E-01
Age(61-80Yrs)-vs-Age(81-100Yrs)	6.74E-01
**Weight**	
Normal-vs-NormalWeight	**3.42E-02**
Normal-vs-ExtremeWeight	7.60E-01
Normal-vs-Obese	1.94E-01
Normal-vs-ExtremeObese	6.19E-01
NormalWeight-vs-ExtremeWeight	6.53E-02
NormalWeight-vs-Obese	3.95E-01
NormalWeight-vs-ExtremeObese	5.59E-01
ExtremeWeight-vs-Obese	2.06E-01
ExtremeWeight-vs-ExtremeObese	6.82E-01
Obese-vs-ExtremeObese	2.90E-01
**Grade**	
Normal-vs-Grade1	8.34E-01
Normal-vs-Grade2	2.58E-01
Normal-vs-Grade3	**1.62E-02**
Grade1-vs-Grade2	1.43E-01
Grade1-vs-Grade3	3.37E-01
Grade2-vs-Grade3	**<1E-12**
**Tumor histology**	
Normal-vs-Lepidic adenocarcinoma	2.52E-01

Normal-vs-Papillary adenocarcinoma	2.16E-01
Normal-vs-Squamous cell carcinoma	NA
Normal-vs-Adenocarcinoma	1.42E-01
Normal-vs-Colloid adenocarcinoma	NA
Normal-vs-Acinar adenocarcinoma	1.18E-01
Normal-vs-Solid adenocarcinoma	**4.25E-03**
Normal-vs-Other	1.61E-01
Lepidic adenocarcinoma-vs-Papillary adenocarcinoma	8.78E-02
Lepidic adenocarcinoma-vs-Squamous cell carcinoma	NA
Lepidic adenocarcinoma-vs-Adenocarcinoma	9.06E-02
Lepidic adenocarcinoma-vs-Colloid adenocarcinoma	NA
Lepidic adenocarcinoma-vs-Acinar adenocarcinoma	7.81E-02
Lepidic adenocarcinoma-vs-Solid adenocarcinoma	1.38E-01
Lepidic adenocarcinoma-vs-Other	**4.32E-02**
Papillary adenocarcinoma-vs-Squamous cell carcinoma	NA
Papillary adenocarcinoma-vs-Adenocarcinoma	7.97E-01
Papillary adenocarcinoma-vs-Colloid adenocarcinoma	NA
Papillary adenocarcinoma-vs-Acinar adenocarcinoma	9.18E-01
Papillary adenocarcinoma-vs-Solid adenocarcinoma	9.72E-01
Papillary adenocarcinoma-vs-Other	7.71E-01
Squamous cell carcinoma-vs-Adenocarcinoma	NA
Squamous cell carcinoma-vs-Colloid adenocarcinoma	NA
Squamous cell carcinoma-vs-Acinar adenocarcinoma	NA
Squamous cell carcinoma-vs-Solid adenocarcinoma	NA
Squamous cell carcinoma-vs-Other	NA
Adenocarcinoma-vs-Colloid adenocarcinoma	NA
Adenocarcinoma-vs-Acinar adenocarcinoma	8.72E-01
Adenocarcinoma-vs-Solid adenocarcinoma	7.48E-01
Adenocarcinoma-vs-Other	6.15E-01
Colloid adenocarcinoma-vs-Acinar adenocarcinoma	NA
Colloid adenocarcinoma-vs-Solid adenocarcinoma	NA
Colloid adenocarcinoma-vs-Other	NA
Acinar adenocarcinoma-vs-Solid adenocarcinoma	9.20E-01
Acinar adenocarcinoma-vs-Other	7.05E-01
Solid adenocarcinoma-vs-Other	7.17E-01
**HIPPO pathway status**	
Normal-vs-HIPPO pathway altered	**4.81E-02**
Normal-vs-Others	9.10E-02
HIPPO pathway altered-vs-Others	2.73E-01
**WNT pathway status**	
Normal-vs-WNT pathway altered	**4.54E-02**
Normal-vs-Others	9.71E-02
WNT pathway altered-vs-Others	6.09E-01
**mTOR pathway status**	
Normal-vs-mTOR pathway altered	**4.06E-02**
Normal-vs-Others	1.17E-01
mTOR pathway altered-vs-Others	4.76E-01
**NRF2 pathway status**	
Normal-vs-NRF2 pathway altered	1.32E-01
Normal-vs-Others	**3.54E-02**
NRF2 pathway altered-vs-Others	5.18E-01
**RTK pathway status**	
Normal-vs-RTK pathway altered	3.86E-02
Normal-vs-Others	1.31E-01
RTK pathway altered-vs-Others	7.20E-01
**p53/Rb- related pathway status**	
Normal-vs-p53/Rb-related pathway altered	**1.45E-02**
Normal-vs-Others	4.92E-01

p53/Rb-related pathway altered-vs-Others	7.37E-01
**SWI-SNF complex status**	
Normal-vs-SWI-SNF complex altered	**2.22E-02**
Normal-vs-Others	2.90E-01
SWI-SNF complex altered-vs-Others	9.90E-01
**MYC/MYCN ststus**	
Normal-vs-MYC/MYCN altered	**4.88E-02**
Normal-vs-Others	9.27E-02
MYC/MYCN altered-vs-Others	5.95E-01
**Chromatin modifer ststus**	
Normal-vs-Chromatin Modifier altered	**9.62E-03**
Normal-vs-Others	7.45E-01
Chromatin Modifier altered-vs-Others	4.77E-01

Bold numbers indicate statistical significance, *P⩽0.05, #P⩽0.001, △P⩽0.0001
