# Supplementary material for: Ginsenosides, potential TMPRSS2 inhibitors, a trade-off between the therapeutic combination for anti-PD-1 immunotherapy and the treatment of COVID-19 infection of LUAD patients
Source: Front Pharmacol. 2023 Mar 13;14:1085509. doi: 10.3389/fphar.2023.1085509 (PMC10040610; doi:10.3389/fphar.2023.1085509)
Supplement: Supplementary file 10 [file DataSheet1.PDF]

SI-Table 1. Relation between TMPRSS2 expression and patient progonsis of different cancer in Prognoscan database

CANCER TYPE	DATASET	ENDPOINT	N	HR [95% CI-low CI-upp]	COX P-VALUE
Bladder Cancer	GSE5287	Overall Survival	30	0.92 [0.55 - 1.54]	0.749943
	GSE5287	Overall Survival	30	0.47 [0.09 - 2.39]	0.364789
	GSE13507	Overall Survival	165	1.03 [0.87 - 1.21]	0.737569
	GSE13507	Disease Specific Survival	165	1.02 [0.80 - 1.30]	0.84976
Blood Cancer	GSE12417-GPL96	Overall Survival	163	1.41 [0.25 - 8.04]	0.695609
	GSE12417-GPL570	Overall Survival	79	0.76 [0.10 - 5.83]	0.793248
	GSE5122	Overall Survival	58	1.41 [0.95 - 2.09]	0.092833
	E-TABM-346	Overall Survival	53	1.75 [0.94 - 3.27]	0.079079
	GSE16131-GPL96	Overall Survival	180	1.27 [0.92 - 1.77]	0.146694
	GSE16131-GPL97	Overall Survival	180	1.60 [0.80 - 3.19]	0.179471
	GSE2658	Disease Specific Survival	559	0.77 [0.50 - 1.20]	0.248097
	GSE2658	Disease Specific Survival	559	0.66 [0.45 - 0.96]	**0.031734**
	GSE2658	Disease Specific Survival	559	0.86 [0.66 - 1.11]	0.240611
	GSE2658	Disease Specific Survival	559	0.75 [0.55 - 1.03]	0.07429
Brain Cancer	GSE4271-GPL96	Overall Survival	77	1.30 [0.90 - 1.87]	0.161958
	GSE7696	Overall Survival	70	1.61 [0.37 - 7.12]	0.526721
	MGH-glioma	Overall Survival	50	0.36 [0.04 - 3.73]	0.393048
	GSE4412-GPL96	Overall Survival	74	1.59 [1.05 - 2.41]	**0.027172**
	GSE4412-GPL96	Overall Survival	74	0.82 [0.52 - 1.29]	0.399286
	GSE4412-GPL97	Overall Survival	74	3.41 [1.08 - 10.72]	**0.036103**
Breast Cancer	GSE19615	Distant Metastasis Free Survival	115	1.71 [0.63 - 4.63]	0.29559
	GSE19615	Distant Metastasis Free Survival	115	1.86 [0.93 - 3.73]	0.078471
	GSE19615	Distant Metastasis Free Survival	115	3.34 [0.58 - 19.36]	0.178864
	GSE7849	Disease Free Survival	76	0.77 [0.35 - 1.70]	0.516344
	GSE12276	Relapse Free Survival	204	1.04 [0.90 - 1.20]	0.567325
	GSE12276	Relapse Free Survival	204	1.11 [0.95 - 1.30]	0.201859
	GSE12276	Relapse Free Survival	204	1.10 [1.00 - 1.21]	**0.047954**
	GSE12276	Relapse Free Survival	204	1.04 [0.94 - 1.15]	0.436271
	GSE6532-GPL570	Relapse Free Survival	87	0.84 [0.42 - 1.69]	0.620943
	GSE6532-GPL570	Relapse Free Survival	87	13.96 [1.54 - 126.26]	**0.018975**
	GSE6532-GPL570	Distant Metastasis Free Survival	87	13.96 [1.54 - 126.26]	**0.018975**
	GSE6532-GPL570	Relapse Free Survival	87	1.36 [0.42 - 4.35]	0.605253
	GSE6532-GPL570	Distant Metastasis Free Survival	87	0.84 [0.42 - 1.69]	0.620943
	GSE12093	Distant Metastasis Free Survival	136	0.71 [0.41 - 1.25]	0.239304
	GSE12093	Distant Metastasis Free Survival	136	0.86 [0.54 - 1.35]	0.500619
	GSE11121	Distant Metastasis Free Survival	200	1.07 [0.87 - 1.32]	0.527097
	GSE11121	Distant Metastasis Free Survival	200	1.51 [1.00 - 2.30]	0.052797
	GSE1378	Relapse Free Survival	60	1.24 [0.90 - 1.69]	0.183329
	GSE1379	Relapse Free Survival	60	1.24 [0.85 - 1.80]	0.263382
	GSE9893	Overall Survival	155	1.48 [1.09 - 1.99]	**0.010851**
	GSE2034	Distant Metastasis Free Survival	286	1.07 [0.89 - 1.29]	0.487747
	GSE2034	Distant Metastasis Free Survival	286	0.80 [0.59 - 1.07]	0.129642
	GSE1456-GPL96	Relapse Free Survival	159	1.10 [0.85 - 1.42]	0.460496
	GSE1456-GPL97	Overall Survival	159	1.08 [0.61 - 1.91]	0.800692
	GSE1456-GPL97	Relapse Free Survival	159	1.08 [0.61 - 1.91]	0.792077
	GSE2990	Relapse Free Survival	125	0.84 [0.53 - 1.34]	0.470657
	GSE2990	Distant Metastasis Free Survival	54	0.77 [0.41 - 1.44]	0.412069
	GSE7390	Overall Survival	198	1.25 [0.97 - 1.61]	0.079629
	GSE7390	Distant Metastasis Free Survival	198	1.05 [0.92 - 1.21]	0.463641
	GSE7390	Overall Survival	198	1.09 [0.94 - 1.26]	0.247606
	GSE7390	Relapse Free Survival	198	1.29 [1.06 - 1.57]	**0.011107**
	GSE7390	Distant Metastasis Free Survival	198	1.21 [0.96 - 1.54]	0.107987
	GSE7390	Relapse Free Survival	198	1.07 [0.95 - 1.20]	0.25009
Colorectal Cancer	GSE12945	Disease Free Survival	51	0.15 [0.00 - 42.13]	0.512834
	GSE12945	Overall Survival	62	0.22 [0.01 - 8.76]	0.420054
	GSE17536	Overall Survival	177	0.75 [0.51 - 1.09]	0.128135
	GSE17536	Disease Specific Survival	177	0.73 [0.47 - 1.12]	0.144471
	GSE17536	Disease Specific Survival	177	0.40 [0.17 - 0.91]	**0.030072**
	GSE17536	Overall Survival	177	0.84 [0.65 - 1.08]	0.17047
	GSE17536	Disease Free Survival	145	0.85 [0.58 - 1.24]	0.401952
	GSE17536	Disease Specific Survival	177	0.64 [0.19 - 2.22]	0.485353
	GSE17536	Overall Survival	177	0.39 [0.19 - 0.81]	**0.010994**
	GSE17536	Disease Free Survival	145	0.50 [0.10 - 2.41]	0.385962
	GSE14333	Disease Free Survival	226	0.76 [0.60 - 0.97]	**0.027629**
	GSE14333	Disease Free Survival	226	0.79 [0.59 - 1.06]	0.111274
	GSE14333	Disease Free Survival	226	0.79 [0.65 - 0.95]	**0.013198**
	GSE14333	Disease Free Survival	226	0.82 [0.65 - 1.02]	0.07555
	GSE17537	Overall Survival	55	0.76 [0.33 - 1.72]	0.507041
	GSE17537	Disease Specific Survival	49	0.20 [0.01 - 5.07]	0.32617
Eye Cancer	GSE22138	Distant Metastasis Free Survival	63	0.39 [0.03 - 4.50]	0.451075
	GSE22138	Distant Metastasis Free Survival	63	0.00 [0.00 - 2731.33]	0.201991
Head and neck cancer	GSE2837	Relapse Free Survival	28	0.00 [0.00 - 44.21]	0.217049
	GSE2837	Relapse Free Survival	28	1.21 [0.81 - 1.80]	0.357678
	GSE2837	Relapse Free Survival	28	0.79 [0.32 - 1.93]	0.598228
	GSE2837	Relapse Free Survival	28	0.01 [0.00 - 17.82]	0.243781
Lung Cancer	jacob-00182-CANDF	Overall Survival	82	0.59 [0.23 - 1.55]	0.283878
	jacob-00182-HLM	Overall Survival	79	0.75 [0.57 - 0.99]	**0.041999**
	jacob-00182-MSK	Overall Survival	104	0.77 [0.58 - 1.04]	0.084167
	jacob-00182-MSK	Overall Survival	104	0.35 [0.13 - 0.94]	**0.036231**
	GSE13213	Overall Survival	117	0.68 [0.51 - 0.89]	**0.006146**
	GSE31210	Overall Survival	204	0.78 [0.60 - 1.03]	0.077943
	GSE31210	Relapse Free Survival	204	0.79 [0.62 - 1.02]	0.073799
	GSE31210	Relapse Free Survival	204	0.77 [0.63 - 0.93]	**0.007007**
	GSE31210	Overall Survival	204	0.59 [0.41 - 0.86]	**0.005819**
	GSE31210	Relapse Free Survival	204	0.59 [0.44 - 0.79]	**0.000359**
	GSE31210	Overall Survival	204	0.58 [0.44 - 0.76]	**0.000116**
	GSE31210	Relapse Free Survival	204	0.59 [0.48 - 0.73]	**0.000001**
	jacob-00182-UM	Overall Survival	178	0.59 [0.33 - 1.07]	0.081244
	jacob-00182-UM	Overall Survival	178	0.77 [0.62 - 0.95]	**0.014318**
	GSE8894	Relapse Free Survival	138	1.00 [0.89 - 1.13]	0.980211
	GSE8894	Relapse Free Survival	138	4.15 [0.33 - 51.67]	0.269133
	GSE4573	Overall Survival	129	0.83 [0.70 - 0.99]	**0.042151**
	GSE4573	Overall Survival	129	1.01 [0.76 - 1.32]	0.96832
Ovarian cancer	GSE9891	Overall Survival	278	1.21 [0.40 - 3.65]	0.736446
	GSE9891	Overall Survival	278	0.37 [0.17 - 0.80]	**0.012312**
	DUKE-OC	Overall Survival	133	5.41 [1.68 - 17.48]	**0.004736**
	DUKE-OC	Overall Survival	133	0.46 [0.14 - 1.47]	0.190217
	GSE26712	Disease Free Survival	185	1.77 [1.07 - 2.93]	**0.025541**
	GSE26712	Disease Free Survival	185	0.77 [0.43 - 1.38]	0.38326
	GSE26712	Overall Survival	185	1.78 [1.03 - 3.07]	**0.039355**
	GSE14764	Overall Survival	80	1.41 [0.90 - 2.20]	0.132685
Skin cancer	GSE19234	Overall Survival	38	2.21 [0.62 - 7.85]	0.218488
	GSE19234	Overall Survival	38	1.26 [0.71 - 2.25]	0.434009
Soft tissue cancer	GSE30929	Distant Recurrence Free Survival	140	0.08 [0.01 - 0.63]	**0.016603**

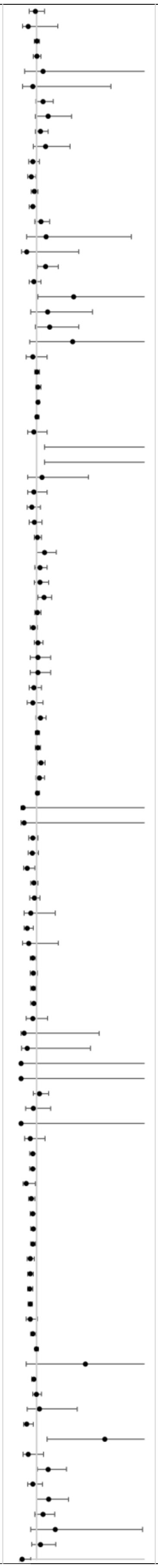

Bold numbers indicate statistical significance.
