# Supplementary material for: Ginsenosides, potential TMPRSS2 inhibitors, a trade-off between the therapeutic combination for anti-PD-1 immunotherapy and the treatment of COVID-19 infection of LUAD patients
Source: Front Pharmacol. 2023 Mar 13;14:1085509. doi: 10.3389/fphar.2023.1085509 (PMC10040610; doi:10.3389/fphar.2023.1085509)
Supplement: Supplementary file 11 [file DataSheet5.PDF]

SI-Table 5. Relation between promoter methylation levels of TMPRSS2 and the clinicopathological parameters in LUAD and LUSC

LUAD		LUSC	
**Sample types**		**Sample types**	
**Comparison**	**Statistical significance**	**Comparison**	**Statistical significance**
Normal-vs-Primary	**1.62E-12**	Normal-vs-Primary	**<1E-12**
**Individual cancer stages**		**Individual cancer stages**	
**Comparison**	**Statistical significance**	**Comparison**	**Statistical significance**
Normal-vs-Stage1	**2.00E-15**	Normal-vs-Stage1	**<1E-12**
Normal-vs-Stage2	**6.50E-10**	Normal-vs-Stage2	**1.62E-12**
Normal-vs-Stage3	**3.19E-06**	Normal-vs-Stage3	**3.11E-12**
Normal-vs-Stage4	**2.76E-02**	Normal-vs-Stage4	1.03E-01
Stage1-vs-Stage2	4.14E-01	Stage1-vs-Stage2	**4.52E-02**
Stage1-vs-Stage3	8.04E-01	Stage1-vs-Stage3	7.37E-01
Stage1-vs-Stage4	4.60E-01	Stage1-vs-Stage4	7.77E-01
Stage2-vs-Stage3	7.21E-01	Stage2-vs-Stage3	2.79E-01
Stage2-vs-Stage4	6.17E-01	Stage2-vs-Stage4	8.60E-01
Stage3-vs-Stage4	5.27E-01	Stage3-vs-Stage4	8.70E-01
**Patient's Race**		**Patient's Race**	
**Comparison**	**Statistical significance**	**Comparison**	**Statistical significance**
Normal-vs-Caucasian	**1.62E-12**	Normal-vs-Caucasian	**<1E-12**
Normal-vs-AfricanAmerican	**2.20E-04**	Normal-vs-AfricanAmerican	**1.20E-07**
Normal-vs-Asian	9.08E-01	Normal-vs-Asian	**9.80E-03**
Caucasian-vs-AfricanAmerican	2.92E-01	Caucasian-vs-AfricanAmerican	6.33E-01
Caucasian-vs-Asian	**4.41E-04**	Caucasian-vs-Asian	8.71E-01
AfricanAmerican-vs-Asian	**4.54E-03**	AfricanAmerican-vs-Asian	9.22E-01
**Patient's Gender**		**Patient's Gender**	
Comparison	**Statistical significance**	Comparison	**Statistical significance**
Normal-vs-Male	**1.66E-12**	Normal-vs-Male	**1.62E-12**
Normal-vs-Female	**1.63E-12**	Normal-vs-Female	**1.62E-12**
Male-vs-Female	1.97E-01	Male-vs-Female	7.41E-01
**Patient's age**		**Patient's age**	
**Comparison**	**Statistical significance**	**Comparison**	**Statistical significance**
Normal-vs-Age(21-40Yrs)	3.03E-01	Normal-vs-Age(21-40Yrs)	N/A
Normal-vs-Age(41-60Yrs)	**3.87E-12**	Normal-vs-Age(41-60Yrs)	**1.62E-12**
Normal-vs-Age(61-80Yrs)	**1.65E-12**	Normal-vs-Age(61-80Yrs)	**1.62E-12**
Normal-vs-Age(81-100Yrs)	**1.70E-03**	Normal-vs-Age(81-100Yrs)	**2.30E-03**
Age(21-40Yrs)-vs-Age(41-60Yrs)	8.71E-01	Age(21-40Yrs)-vs-Age(41-60Yrs)	N/A
Age(21-40Yrs)-vs-Age(61-80Yrs)	6.66E-01	Age(21-40Yrs)-vs-Age(61-80Yrs)	N/A
Age(21-40Yrs)-vs-Age(81-100Yrs)	9.69E-01	Age(21-40Yrs)-vs-Age(81-100Yrs)	N/A
Age(41-60Yrs)-vs-Age(61-80Yrs)	**2.17E-02**	Age(41-60Yrs)-vs-Age(61-80Yrs)	1.66E-01
Age(41-60Yrs)-vs-Age(81-100Yrs)	6.73E-01	Age(41-60Yrs)-vs-Age(81-100Yrs)	6.97E-02
Age(61-80Yrs)-vs-Age(81-100Yrs)	4.17E-01	Age(61-80Yrs)-vs-Age(81-100Yrs)	1.35E-01
**Patient's smoking habit**		**Patient's smoking habit**	
**Comparison**	**Statistical significance**	**Comparison**	**Statistical significance**
Normal-vs-NonSmoker	**2.25E-05**	Normal-vs-NonSmoker	**9.44E-04**
Normal-vs-Smoker	**6.17E-10**	Normal-vs-Smoker	**1.62E-12**
Normal-vs-ReformedSmoker1	**1.81E-12**	Normal-vs-ReformedSmoker1	**<1E-12**
Normal-vs-ReformedSmoker2	**1.32E-07**	Normal-vs-ReformedSmoker2	**6.43E-13**
NonSmoker-vs-Smoker	**8.84E-03**	NonSmoker-vs-Smoker	8.00E-01
NonSmoker-vs-ReformedSmoker1	**1.19E-02**	NonSmoker-vs-ReformedSmoker1	6.76E-01
NonSmoker-vs-ReformedSmoker2	8.24E-01	NonSmoker-vs-ReformedSmoker2	5.00E-01
Smoker-vs-ReformedSmoker1	5.80E-01	Smoker-vs-ReformedSmoker1	6.90E-01
Smoker-vs-ReformedSmoker2	**1.01E-02**	Smoker-vs-ReformedSmoker2	4.31E-01
ReformedSmoker1-vs-ReformedSmoker2	**1.27E-02**	ReformedSmoker1-vs-ReformedSmoker2	6.15E-01
**Nodal Metastasis status**		**Nodal Metastasis status**	
**Comparison**	**Statistical significance**	**Comparison**	**Statistical significance**
Normal-vs-N0	**1.62E-12**	Normal-vs-N0	**1.62E-12**
Normal-vs-N1	**1.10E-07**	Normal-vs-N1	**1.62E-12**
Normal-vs-N2	**2.29E-05**	Normal-vs-N2	**6.93E-08**
Normal-vs-N3	N/A	N0-vs-N1	9.72E-02
N0-vs-N1	4.90E-01	N0-vs-N2	7.80E-01
N0-vs-N2	9.67E-01	N1-vs-N2	2.41E-01
N0-vs-N3	N/A		
N1-vs-N2	6.60E-01		
N1-vs-N3	N/A		
N2-vs-N3	N/A		
**TP53 Mutant**		**TP53 Mutant**	
**Comparison**	**Statistical significance**	**Comparison**	**Statistical significance**
Normal-vs-TP53 Mutant	**1.63E-12**	Normal-vs-TP53 Mutant	**<1E-12**
Normal-vs-TP53 NonMutant	**1.67E-12**	Normal-vs-TP53 NonMutant	**<1E-12**
TP53 Mutant-vs-TP53 NonMutant	3.28E-01	TP53 Mutant-vs-TP53 NonMutant	5.27E-02

Bold numbers indicate statistical significance, *P≤0.05, #P≤0.001, △P≤0.0001
